# Supplementary material for: Identification of Freezing-Responsive microRNAs and Their Targets in Chinese Jujube by Small RNA and Degradome Sequencing
Source: Int J Mol Sci. 2026 Sep 4;27(17):7912. doi: 10.3390/ijms27177912 (PMC13566890; doi:10.3390/ijms27177912)
Supplement: Supplementary file 1 [file ijms-27-07912-s001.zip › Supplementary Material S2/novel_miR_14.pdf]

Provisional ID	:	novel_miR_14
Score total	:	0.5
Score for star read(s)	:	-1.3
Score for read counts	:	0
Score for mfe	:	0.2
Score for randfold	:	1.6
Score for cons. seed	:	
Total read count	:	140253
Mature read count	:	140253
Loop read count	:	0
Star read count	:	0

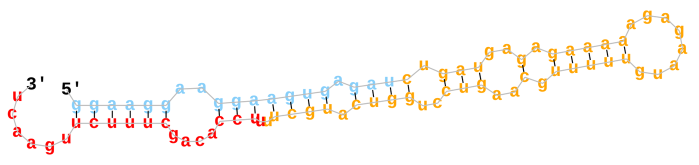

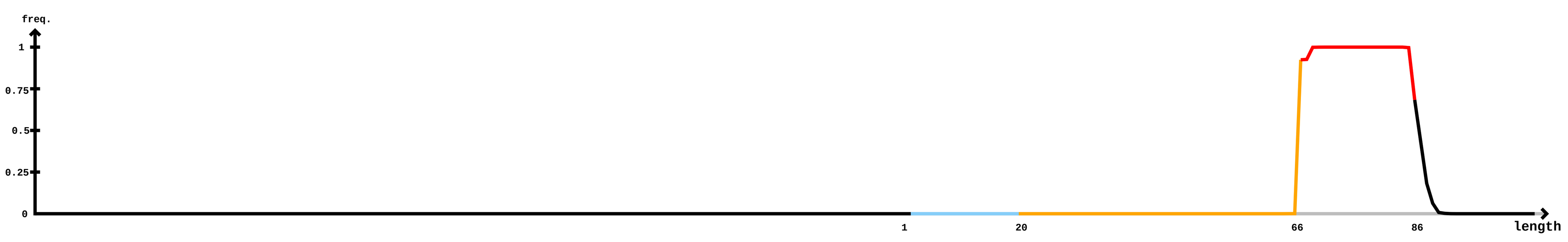

Star Mature

```
5'- uugcuaaaacccacaaauuacuagaaaaaaaaaaaaaguaaagaaaaaaaaaaaaaaaaacuuacuacuacuacuaguaguauaucuucuaauuugcuggaaaaggcacaccaaagagaaagaaauuaaaccaaggaagggaggaaggaaggaaggaagugagaucugaugagagaaaaagagaauguuuuugcaaguccuggucaugcuuuuccacagcuuucuugaacuuucuuucuuucuguuuagucu -3'
    .((((.....(((((((((..(((.........(((..................)))((((((((....))))))))..)))..))))))).)))....))))...............((((((..(((((((((((((..(((((((..((((((.(((((.((.((....((((....)))).....)))).)))).)..))).)))..)))))))...))))))))))))))))))))..
```

exp	reads	mm	sample
aaaaaaGaaaaaguaaag	1	1	JT-1
aaaaagGaaagaaaaaaaaaa	5	1	LCK-3
uuuuccacagcuuucuugaac	3	0	LT-3
uuuuccacagcuuucuugaac	3	0	JT-3
uuuuccacagcuuucuugaac	2	0	JT-2
uuuuccacagcuuucuugaac	7	0	JCK-3
uuuuccacagcuuucuugaac	6	0	JCK-2
uuuuccacagcuuucuugaac	4	0	JCK-1
uuuuccacagcuuucuugaac	3	0	LT-1
uuuuccacagcuuucuugaac	3	0	LCK-3
uuuuccacagcuuucuugaacu	1	0	JCK-3
uuuuccacagcuuucuugaacu	4	0	JCK-2
uNuuccacagcuuucuugaacu	1	1	JCK-3
uuuccacagcuuucuuga	2	0	JCK-1
uuuccacagcuuucuugaacu	1	0	JCK-2
Auuccacagcuuucuugaacu	1	1	JCK-3
uuuccacagcuuucuugaacu	1	0	LT-1
Guuccacagcuuucuugaacu	2	1	LT-3
uuuccacagcuuucuugaacu	1	0	JCK-3
Auuccacagcuuucuugaacu	2	1	JCK-2
uuuccacagcuuucuugaacu	2	0	LCK-3
Cuuccacagcuuucuugaacuu	1	1	LT-2
Cuuccacagcuuucuugaacuu	3	1	JCK-2
Cuuccacagcuuucuugaacuu	2	1	JCK-1
Cuuccacagcuuucuugaacuu	3	1	LT-1
Cuuccacagcuuucuugaacuu	3	1	LT-3
Cuuccacagcuuucuugaacuuu	3	1	LCK-3
Cuuccacagcuuucuugaacuuu	1	1	LT-3
Cuuccacagcuuucuugaacuuu	1	1	LT-1
Cuuccacagcuuucuugaacuuu	2	1	JCK-1
uuccacagcuuucuugaa	52	0	LT-3
uuccacagcuuucuugaU	4	1	LCK-3
uuccacagcuuucuugaU	1	1	JCK-2
uuccacagcuuucuugaa	15	0	JCK-2

Star Mature

uugcuaaaacccacaaauuacuagaaaaaaaaaaaaguaaagaaaaaaaaaaaaaaaacuuacuacuacuacuaguaguauaucuucuaauuugcuggaaaaggcacaccaaagagaaagaaauuaaaccaaggaagggaggaaggaaggaaggaagugagaucugaugagagaaaaagagaauguuuuugcaaguccuggucaugcuuuuccacagcuuucuugaacuuucuuucuuucuguuuagucu

...uuccacagcuuucuugaU...	1	1	JCK-1
...uuccacagcuuucuugaa...	53	0	LCK-2
...uuccacagcuuucuuUaa...	1	1	LT-1
...uuccacagcuuucuugaU...	3	1	LT-2
...uuccacagcuuucuugaC...	1	1	JCK-3
...uuccacagcuuucuGgaa...	1	1	LCK-1
...uuccacagcuuucuugCa...	1	1	LT-2
...uuccacagcuuucuugaa...	43	0	LCK-3
...uuccacagcuuucuugaa...	31	0	LT-1
...uuccacagcuuucuugaU...	14	1	LT-3
...uuccacagcuuucuugUa...	5	1	JT-1
...uuccacagcuuucuugUa...	4	1	LT-3
...uuccacagcuuucuugaa...	43	0	LT-2
...uuccaNagcuuucuugaa...	1	1	LCK-1
...uuccacagcuuucuugaa...	5	0	JT-2
...uuccacagcuuucuugaa...	46	0	LCK-1
...uuccacagcuuucuugaa...	15	0	JT-3
...uuccacagcuuucuugaC...	4	1	LT-2
...uuccacagcuuucuugaa...	9	0	JCK-3
...uuccacagcuuucuugUa...	19	1	LCK-3
...uuccacagcuuucuugaU...	11	1	LT-1
...uuccacagcuuucuugaC...	1	1	LT-3
...uuccacagcuuucuugUa...	15	1	LT-2
...uuccacagcuuucuugUa...	1	1	LT-1
...uuccacagcuuucuugUa...	2	1	JCK-3
...uuccacagcuuucuugaa...	16	0	JCK-1
...uuccacagcuuucuugGa...	1	1	LCK-2
...uuccacagcuuucuugaaU...	28	1	JCK-3
...uucUacagcuuucuugaac...	4	1	JCK-2
...uuccacagUuuucuugaac...	5	1	LT-1
...uuccacagcuuucuugaCc...	8	1	LCK-3
...uuccacagcuuucuugaaU...	24	1	LCK-2
...uuccacagcuuucuugaUc...	2	1	LT-3
...uuccacGgcuuucuugaac...	2	1	JCK-2
...uuccacagcuuucuugaGc...	1	1	LT-3
...uuccacagcuGucuugaac...	1	1	LT-1
...uuccacagcuuucCugaac...	1	1	JCK-3
...uuccacagcuuCcuugaac...	3	1	JCK-2
...uCccacagcuuucuugaac...	1	1	JCK-1
...uuccaUagcuuucuugaac...	2	1	LT-3
...uuccacagcuuucuGgaac...	2	1	LCK-1
...uuccacagcCuucuugaac...	1	1	JCK-1
...uuccacagcuuucuugaCc...	1	1	JT-1
...uuccacagcuuucuugaaU...	98	1	LT-3
...uuccCcagcuuucuugaac...	13	1	LCK-3
...uuccacaAcuuucuugaac...	1	1	LT-3
...uuccCcagcuuucuugaac...	16	1	LT-3
...uuccacagcuuucuuUaac...	1	1	JT-1
...uuccacagcuuucuCgaac...	1	1	LT-1
...uuccacagcuuucCugaac...	2	1	LT-1
...uuccacagcuuucNugaac...	6	1	LCK-1
...uuccacagcuuucuugaac...	1739	0	LCK-2
...uuccCcagcuuucuugaac...	1	1	LCK-2
...uuccacagcuuucuugaaU...	24	1	JCK-1
...uuccacagcuuucGugaac...	3	1	LCK-1
...uuNcacagcuuucuugaac...	14	1	LT-2
...uuccacagcuuucuugaac...	6061	0	LT-1
...uuccGcagcuuucuugaac...	1	1	LT-1
...uucUacagcuuucuugaac...	2	1	JCK-1
...uuUcacagcuuucuugaac...	2	1	JCK-2
...uuccacaUcuuucuugaac...	2	1	JCK-2
...uuccacagcuuucuugaaU...	103	1	LCK-3
...Cuccacagcuuucuugaac...	3	1	LT-3
...uuccacagcuuucuugCac...	5	1	LT-2
...uuccCcagcuuucuugaac...	33	1	LT-2
...uuccaAagcuuucuugaac...	1	1	JCK-1
...uucNacagcuuucuugaac...	1	1	JCK-1
...uuccacagcuuuAuugaac...	2	1	LCK-3
...uucUacagcuuucuugaac...	1	1	JCK-3
...uuccacagcuuuUuugaac...	1	1	LCK-2

```
                                                                                                                                                                                                                   Star                                                                                 Mature
uugcuaaaacccacaaauuacuagaaaaaaaaaaaaaguaaagaaaaaaaaaaaaaaaaacuuacuacuacuacuaguaguauaucuucuaauuugcuggaaaaggcacaccaaagagaaagaaauuaaaccaaggaagggaggaaggaaggaaggaagugagaucugaugagagaaaaagagaauguuuuugcaaguccuggucaugcuuuuccacagcuuucuugaacuuucuuucuuucuguuuagucu
...................................................................................................................................................................................................................uuccacagcuuuAuugaac......................   1      1     LT-2
...................................................................................................................................................................................................................uuccaUagcuuucuugaac......................   1      1     JCK-2
...................................................................................................................................................................................................................uuAcacagcuuucuugaac......................   1      1     LT-3
...................................................................................................................................................................................................................uuUcacagcuuucuugaac......................   6      1     JCK-1
...................................................................................................................................................................................................................uuccacagcuuucuugaac......................   8415   0     LCK-3
...................................................................................................................................................................................................................uuccacagcuuucuugaac......................   5694   0     LT-2
...................................................................................................................................................................................................................uuccacagcuuucuugGac......................   1      1     JCK-3
...................................................................................................................................................................................................................uNccacagcuuucuugaac......................   2      1     JCK-3
...................................................................................................................................................................................................................uuccacagcuuucuugaaU......................   26     1     LCK-1
...................................................................................................................................................................................................................uuccacagcCuucuugaac......................   7      1     LT-1
...................................................................................................................................................................................................................uuccacagcuuucuugaac......................   8638   0     LT-3
...................................................................................................................................................................................................................uuccCcagcuuucuugaac......................   2      1     LCK-1
...................................................................................................................................................................................................................uuccacagcuCucuugaac......................   2      1     JCK-2
...................................................................................................................................................................................................................uuccaUagcuuucuugaac......................   1      1     LT-2
...................................................................................................................................................................................................................uuccacagcuuucuuAaac......................   1      1     JCK-1
...................................................................................................................................................................................................................Cuccacagcuuucuugaac......................   1      1     JCK-3
...................................................................................................................................................................................................................uuccacagcuuucuugaUc......................   3      1     LT-2
...................................................................................................................................................................................................................uuccacagcuuucuugaac......................   3571   0     JCK-2
...................................................................................................................................................................................................................uuUcacagcuuucuugaac......................   1      1     LT-1
...................................................................................................................................................................................................................uNccacagcuuucuugaac......................   1      1     LCK-2
...................................................................................................................................................................................................................uuccCcagcuuucuugaac......................   2      1     JCK-3
...................................................................................................................................................................................................................uuccacagcuuNcuugaac......................   2      1     JCK-1
...................................................................................................................................................................................................................uuccacagcuuucuugaGc......................   1      1     LCK-3
...................................................................................................................................................................................................................uuccacagcCuucuugaac......................   1      1     LT-2
...................................................................................................................................................................................................................uuccacCgcuuucuugaac......................   9      1     JCK-2
...................................................................................................................................................................................................................uuccacagcuuuUuugaac......................   4      1     JCK-1
...................................................................................................................................................................................................................uuccacagcuuucuugaCc......................   5      1     LT-1
...................................................................................................................................................................................................................uuccacagcuuuNuugaac......................   1      1     LCK-1
...................................................................................................................................................................................................................uuccaAagcuuucuugaac......................   1      1     JCK-2
...................................................................................................................................................................................................................uuccaAagcuuucuugaac......................   1      1     LT-2
...................................................................................................................................................................................................................uuccacCgcuuucuugaac......................   1      1     JT-2
...................................................................................................................................................................................................................uNccacagcuuucuugaac......................   5      1     JCK-2
...................................................................................................................................................................................................................uuccacagcuuucuugCac......................   5      1     JCK-2
...................................................................................................................................................................................................................uuGcacagcuuucuugaac......................   3      1     LT-2
...................................................................................................................................................................................................................uuccacaAcuuucuugaac......................   5      1     LT-1
...................................................................................................................................................................................................................uuccacagcuuucuNgaac......................   4      1     LCK-1
...................................................................................................................................................................................................................uuccacCgcuuucuugaac......................   10     1     LT-2
...................................................................................................................................................................................................................uuccacagUuuucuugaac......................   1      1     LT-3
...................................................................................................................................................................................................................uCccacagcuuucuugaac......................   1      1     LCK-1
...................................................................................................................................................................................................................uuccacagcuuucuugaaU......................   53     1     JCK-2
...................................................................................................................................................................................................................uuccacagAuuucuugaac......................   1      1     LT-2
...................................................................................................................................................................................................................uuccacagcuuuUuugaac......................   8      1     JCK-2
...................................................................................................................................................................................................................uuccacagcuuCcuugaac......................   3      1     JCK-1
...................................................................................................................................................................................................................uuccCcagcuuucuugaac......................   12     1     LT-1
...................................................................................................................................................................................................................uuccacagcuuucuugCac......................   4      1     LT-1
...................................................................................................................................................................................................................Cuccacagcuuucuugaac......................   1      1     LT-2
...................................................................................................................................................................................................................uuccacagcuuucuugaac......................   2180   0     JCK-1
...................................................................................................................................................................................................................uuccacCgcuuucuugaac......................   2      1     JT-1
...................................................................................................................................................................................................................uuUcacagcuuucuugaac......................   1      1     JCK-3
...................................................................................................................................................................................................................uuccaNagcuuucuugaac......................   3      1     LCK-1
...................................................................................................................................................................................................................uuccacagcuuucuugUac......................   1      1     LT-3
...................................................................................................................................................................................................................uuccacagcuuCcuugaac......................   2      1     JCK-3
...................................................................................................................................................................................................................uuccacagcuuucuugaCc......................   1      1     JCK-3
...................................................................................................................................................................................................................uuccacagcuuucCugaac......................   2      1     LCK-3
...................................................................................................................................................................................................................uuccacagcuuucuugaUc......................   2      1     JCK-1
...................................................................................................................................................................................................................uuccacagcuuucuugCac......................   3      1     JT-1
...................................................................................................................................................................................................................uuccacagcCuucuugaac......................   5      1     LT-3
...................................................................................................................................................................................................................uuccacagcuuucuugaCc......................   1      1     LCK-1
...................................................................................................................................................................................................................uuccacagcuCucuugaac......................   1      1     LT-1
...................................................................................................................................................................................................................Cuccacagcuuucuugaac......................   3      1     LCK-3
...................................................................................................................................................................................................................uuccaAagcuuucuugaac......................   3      1     LT-3
...................................................................................................................................................................................................................uuccacagcuuucuugaCc......................   7      1     JCK-2
...................................................................................................................................................................................................................uuccacagcuuucuugaaU......................   37     1     LT-1
...................................................................................................................................................................................................................uuccacagcuuucuugaac......................   542    0     JT-3
...................................................................................................................................................................................................................uuccacagcuuucuugaUc......................   10     1     LCK-3
...................................................................................................................................................................................................................uuUcacagcuuucuugaac......................   1      1     LT-3
...................................................................................................................................................................................................................Cuccacagcuuucuugaac......................   4      1     LT-1
...................................................................................................................................................................................................................uuccacagcuuucuugCac......................   1      1     LCK-2
...................................................................................................................................................................................................................uuccacagcCuucuugaac......................   1      1     JCK-2
...................................................................................................................................................................................................................uuccacaAcuuucuugaac......................   1      1     JCK-3
```

Star / Mature			
uugcuaaaacccacaaauuacuagaaaaaaaaaaaaaguaaagaaaaaaaaaaaaaaaacuuacuacuacuacuaguaguauaucuucuaauuugcuggaaaaggcacaccaaagagaaagaaauuaaaccaaggaagggaggaaggaaggaaggaagugagaucugaugagagaaaaagagaauguuuuugcaaguccuggucaugcuuuuccacagcuuucuugaacuuucuuucuuucuguuuagucu			
uuccacCgcuuucuugaac	16	1	LT-3
uuccacCgcuuucuugaac	12	1	LT-1
uNccacagcuuucuugaac	1	1	JCK-1
uuccacagcuuucuugaac	1423	0	JT-1
uuccaAagcuuucuugaac	1	1	LCK-3
uuccacagcuuucuugaCc	13	1	LT-2
uuccacagcuuucuugaGc	1	1	JCK-1
uuccacagcuuucuugCac	1	1	LCK-1
uuccCcagcuuucuugaac	4	1	JCK-2
uuccacagcuuucuugUac	3	1	LCK-3
uuccacagcuGucuugaac	3	1	JCK-1
uuNcacagcuuucuugaac	1	1	LCK-1
uuccacCgcuuucuugaac	2	1	LCK-2
uuccacagcuuucuugCac	1	1	JCK-3
uuccacagcuuucuugaac	411	0	JT-2
Cuccacagcuuucuugaac	2	1	JT-1
uuccacagcuuucuugCac	3	1	LCK-3
uuccacagUuuucuugaac	3	1	LT-2
uuccacagcuuCcuugaac	3	1	LCK-3
uuccacagcuuucuGgaac	1	1	JCK-2
uuccacaAcuuucuugaac	1	1	JCK-2
uuccacagcuuucuugCac	11	1	LT-3
uuccacaAcuuucuugaac	2	1	LT-2
uuccacagcuuCcuugaac	2	1	LT-1
uuccacagUuuucuugaac	4	1	LCK-3
uNccacagcuuucuugaac	3	1	JT-1
uuccacagcuuucCugaac	3	1	JCK-2
uuccacCgcuuucuugaac	2	1	JCK-3
uCccacagcuuucuugaac	2	1	LT-3
uuccacCgcuuucuugaac	2	1	JCK-1
uuccacagcuuuUuugaac	6	1	LT-2
uuccacagcuuucCugaac	1	1	LT-2
uuAcacagcuuucuugaac	1	1	JCK-1
uuccacagcuuucuugaac	1676	0	LCK-1
uuccacagcuuucuugaUc	3	1	JT-1
uuccacagcuuucuugCac	2	1	JCK-1
uuccacagcuuucuugaaU	64	1	LT-2
uuccacagcuuucuugaac	2667	0	JCK-3
uucAacagcuuucuugaac	1	1	LT-2
uCccacagcuuucuugaac	1	1	JCK-2
uuccacagcuuucuugaCc	10	1	LT-3
uuccacagcuuuUuugaac	2	1	JCK-3
uuccCcagcuuucuugaac	2	1	JT-1
uuccacaAcuuucuugaac	2	1	LCK-3
uuccacCgcuuucuugaac	11	1	LCK-3
uuccCcagcuuucuugaac	1	1	JCK-1
uuccacagcuuucuuAaac	4	1	LT-2
uuccacagcuuuAuugaac	2	1	LT-1
uuccacagcuuucuugaCc	2	1	LCK-2
uuccCcagcuuucuugaac	1	1	JT-3
uuccacagcuuuUuugaac	3	1	LT-1
uuccacagcuuucuGgaacu	1	1	JT-1
uCccacagcuuucuugaacu	2	1	JCK-2
uuccacagcuuucuugaacC	2	1	JCK-2
uuccacagcCuucuugaacu	1	1	JT-2
uuccacagcuuuAuugaacu	1	1	LT-2
uuccacagcuuucuugaacG	2	1	LT-1
uuccacagcuuucuugaacC	5	1	JCK-3
uuccacGgcuuucuugaacu	1	1	LCK-3
uuccacagcuuucuugCacu	10	1	LCK-3
uuccaAagcuuucuugaacu	1	1	JCK-2
uCccacagcuuucuugaacu	1	1	LCK-3
uuccacagcuuucuugaaUu	154	1	LT-2
uuccacagcuuucuugaaUu	172	1	LT-3
uuccacagcuuucuugaacA	43	1	JCK-3
uuccacagcuuucuugCacu	6	1	LT-3
uuccacagcuuucuCgaacu	1	1	LT-2
uuccacagcuuucuugaCcu	1	1	LCK-2
uuccacagcuuucuCgaacu	1	1	JCK-1
uuccacagcuuucGugaacu	1	1	LCK-1

```
                                                                                                                                                                    Star                                                  Mature
uugcuaaaacccacaaauuacuagaaaaaaaaaaaaaguaaagaaaaaaaaaaaaaaaacuuacuacuacuacuaguaguauaucuucuaauuugcuggaaaaggcacaccaaagagaaagaaauuaaaccaaggaagggaggaaggaaggaaggaagugagaucugaugagagaaaaagagaauguuuuugcaaguccuggucaugcuuuuccacagcuuucuugaacuuucuuucuuucuguuuagucu
..................................................................................................................................................................................................................uuccacagcuuucuugaNcu.....................   2       1   JCK-3
..................................................................................................................................................................................................................uuccacCgcuuucuugaacu.....................   9       1   LCK-3
..................................................................................................................................................................................................................uuccacagcuuucuugaCcu.....................   1       1   JT-2
..................................................................................................................................................................................................................uucUacagcuuucuugaacu.....................   3       1   LT-2
..................................................................................................................................................................................................................uuccCcagcuuucuugaacu.....................   2       1   JT-3
..................................................................................................................................................................................................................uuccacagcCuucuugaacu.....................   5       1   LT-1
..................................................................................................................................................................................................................uuccacagcuuucNugaacu.....................   5       1   LCK-1
..................................................................................................................................................................................................................uuccacagcuuucuugaacC.....................   41      1   LCK-3
..................................................................................................................................................................................................................uuccacagcuuucuugaacu.....................   6887    0   LT-2
..................................................................................................................................................................................................................uuccCcagcuuucuugaacu.....................   20      1   LT-2
..................................................................................................................................................................................................................uuccacagcuuucuugaCcu.....................   1       1   JCK-3
..................................................................................................................................................................................................................uuccacagcuuucuugaacu.....................   4415    0   LT-1
..................................................................................................................................................................................................................uNccacagcuuucuugaacu.....................   2       1   JCK-3
..................................................................................................................................................................................................................uuccacCgcuuucuugaacu.....................   3       1   LCK-2
..................................................................................................................................................................................................................uuccacagcuuucuugaCcu.....................   8       1   LT-3
..................................................................................................................................................................................................................uucNacagcuuucuugaacu.....................   2       1   JCK-1
..................................................................................................................................................................................................................uuccacagcuuucuugaCcu.....................   11      1   LCK-3
..................................................................................................................................................................................................................uuccacagcuuucuuUaacu.....................   1       1   LT-2
..................................................................................................................................................................................................................uuccacCgcuuucuugaacu.....................   1       1   JT-1
..................................................................................................................................................................................................................uuccacagcuuucuuCaacu.....................   1       1   JCK-1
..................................................................................................................................................................................................................uuccacagcuuucCugaacu.....................   1       1   LT-1
..................................................................................................................................................................................................................uuNcacagcuuucuugaacu.....................   2       1   LCK-3
..................................................................................................................................................................................................................uuccacagcuuucuGgaacu.....................   4       1   LCK-1
..................................................................................................................................................................................................................uNccacagcuuucuugaacu.....................   4       1   JCK-1
..................................................................................................................................................................................................................uuccCcagcuuucuugaacu.....................   4       1   JCK-1
..................................................................................................................................................................................................................uuccacagcuuucuGgaacu.....................   1       1   LT-3
..................................................................................................................................................................................................................uuccacagcuuucuugCacu.....................   10      1   LT-2
..................................................................................................................................................................................................................uuccacagcuCucuugaacu.....................   3       1   LT-1
..................................................................................................................................................................................................................uuccacagcuuucuugaaAu.....................   2       1   LT-1
..................................................................................................................................................................................................................uuccacagcuuucuugaUcu.....................   2       1   LT-1
..................................................................................................................................................................................................................uuccacagcuuucuuAaacu.....................   1       1   JCK-1
..................................................................................................................................................................................................................uuccacagcuuucuugaacu.....................   2316    0   LCK-1
..................................................................................................................................................................................................................uuccacagcuuucuugaGcu.....................   3       1   JCK-1
..................................................................................................................................................................................................................uuccacagcuuucuugaacC.....................   39      1   LT-2
..................................................................................................................................................................................................................uuccacagcCuucuugaacu.....................   3       1   JCK-2
..................................................................................................................................................................................................................uuccaAagcuuucuugaacu.....................   1       1   LCK-1
..................................................................................................................................................................................................................uuccacagcuuucuugaUcu.....................   2       1   LT-2
..................................................................................................................................................................................................................uuccacaAcuuucuugaacu.....................   1       1   JT-1
..................................................................................................................................................................................................................uuccacagcuuucuugaaUu.....................   35      1   LCK-2
..................................................................................................................................................................................................................uuccacagUuuucuugaacu.....................   1       1   JT-1
..................................................................................................................................................................................................................uuccacagcCuucuugaacu.....................   1       1   JCK-3
..................................................................................................................................................................................................................uuccacagcuuucuugaacA.....................   11      1   JT-3
..................................................................................................................................................................................................................uuccacagcuuucuugaacG.....................   45      1   LCK-3
..................................................................................................................................................................................................................uuccacagcuCucuugaacu.....................   1       1   JT-1
..................................................................................................................................................................................................................uuccacagcuuucuugaacG.....................   6       1   LT-3
..................................................................................................................................................................................................................uuccacagcuGucuugaacu.....................   1       1   LT-2
..................................................................................................................................................................................................................uuccacagcuuCcuugaacu.....................   2       1   LT-3
..................................................................................................................................................................................................................uuccacagcuuucuuAaacu.....................   1       1   LT-2
..................................................................................................................................................................................................................uuccacagcuuucuugaacN.....................   2       1   JCK-1
..................................................................................................................................................................................................................uuccacagcuuucuugaacA.....................   87      1   LT-1
..................................................................................................................................................................................................................uuccacagcuuCcuugaacu.....................   1       1   JCK-2
..................................................................................................................................................................................................................uuccacagcuuucuugaacu.....................   10755   0   LCK-3
..................................................................................................................................................................................................................uuccacagcuuuAuugaacu.....................   4       1   LCK-3
..................................................................................................................................................................................................................uuccacCgcuuucuugaacu.....................   3       1   JCK-1
..................................................................................................................................................................................................................uCccacagcuuucuugaacu.....................   3       1   JCK-3
..................................................................................................................................................................................................................uuccacagcuuucuugaaUu.....................   24      1   LCK-1
..................................................................................................................................................................................................................uuccacagcuuucuugaCcu.....................   1       1   LT-1
..................................................................................................................................................................................................................uuccacagcuuucuugaacu.....................   2326    0   LCK-2
..................................................................................................................................................................................................................uuUcacagcuuucuugaacu.....................   1       1   JCK-3
..................................................................................................................................................................................................................uuccacagcuuucuugaaUu.....................   45      1   JT-2
..................................................................................................................................................................................................................uuccacagcuGucuugaacu.....................   1       1   JCK-1
..................................................................................................................................................................................................................uuccacaAcuuucuugaacu.....................   4       1   LCK-3
..................................................................................................................................................................................................................uuccacagcuuCcuugaacu.....................   2       1   LT-2
..................................................................................................................................................................................................................uuccaNagcuuucuugaacu.....................   3       1   LCK-1
..................................................................................................................................................................................................................uuccacagcuuucuugaacu.....................   2055    0   JT-1
..................................................................................................................................................................................................................uuccacCgcuuucuugaacu.....................   4       1   LT-1
..................................................................................................................................................................................................................uuccacagcuuucuugaaUu.....................   111     1   JCK-2
..................................................................................................................................................................................................................uuccacagcuuucCugaacu.....................   1       1   LT-3
..................................................................................................................................................................................................................uuccacagcuuucuugCacu.....................   2       1   LCK-1
..................................................................................................................................................................................................................uuccacagcuuucuugaacC.....................   18      1   LT-3
```

```
                                                                                                                                                 Star                                                             Mature
uugcuaaaacccacaaauuacuagaaaaaaaaaaaaaguaaagaaaaaaaaaaaaaaaacuuacuacuacuacuaguaguauaucuucuaauuugcuggaaaaggcacaccaaagagaaagaaauuaaaccaaggaagggaggaaggaaggaaggaagugagaucugaugagagaaaaagagaauguuuuugcaaguccuggucaugcuuuuccacagcuuucuugaacuuucuuucuuucuguuuagucu
..................................................................................................................................................................................................................uuccacagcuuucuugaacA.....................   20     1     JT-1
..................................................................................................................................................................................................................uuccacagcuuucCugaacu.....................   1      1     JT-1
..................................................................................................................................................................................................................uuccacaAcuuucuugaacu.....................   1      1     LT-3
..................................................................................................................................................................................................................uuccacagcuuucuugCacu.....................   10     1     LT-1
..................................................................................................................................................................................................................Cuccacagcuuucuugaacu.....................   1      1     JCK-2
..................................................................................................................................................................................................................Cuccacagcuuucuugaacu.....................   2      1     JCK-3
..................................................................................................................................................................................................................uuccacagcuuucuugaacC.....................   8      1     JT-1
..................................................................................................................................................................................................................Cuccacagcuuucuugaacu.....................   1      1     LT-3
..................................................................................................................................................................................................................uuccacagcuuucuugaCcu.....................   3      1     JCK-2
..................................................................................................................................................................................................................uuccacagcuuucuugaaUu.....................   50     1     JT-3
..................................................................................................................................................................................................................uuccacagcuuuNuugaacu.....................   1      1     LCK-1
..................................................................................................................................................................................................................uuccUcagcuuucuugaacu.....................   1      1     JCK-1
..................................................................................................................................................................................................................uuccCcagcuuucuugaacu.....................   8      1     LT-3
..................................................................................................................................................................................................................uuccacagcuuucuugaCcu.....................   3      1     LCK-1
..................................................................................................................................................................................................................uuccacagcuuucuugaacA.....................   3      1     JT-2
..................................................................................................................................................................................................................uuccacagcuuucuNgaacu.....................   7      1     LCK-1
..................................................................................................................................................................................................................uuNcacagcuuucuugaacu.....................   33     1     LT-2
..................................................................................................................................................................................................................uuccacagcuuucuugaacu.....................   544    0     JT-2
..................................................................................................................................................................................................................Cuccacagcuuucuugaacu.....................   4      1     LCK-3
..................................................................................................................................................................................................................uuccacagcuuucuugaacA.....................   172    1     LT-3
..................................................................................................................................................................................................................uuccacaUcuuucuugaacu.....................   1      1     JCK-1
..................................................................................................................................................................................................................uuccacagcuuucuugCacu.....................   2      1     JCK-3
..................................................................................................................................................................................................................uuccacagcuuuAuugaacu.....................   2      1     LT-3
..................................................................................................................................................................................................................uuccacagUuuucuugaacu.....................   1      1     JCK-2
..................................................................................................................................................................................................................uuccCcagcuuucuugaacu.....................   2      1     JT-1
..................................................................................................................................................................................................................uuccCcagcuuucuugaacu.....................   8      1     LCK-3
..................................................................................................................................................................................................................uuccacagcuuucGugaacu.....................   1      1     LCK-2
..................................................................................................................................................................................................................uuccacagcuuNcuugaacu.....................   1      1     JT-2
..................................................................................................................................................................................................................uuccacagUuuucuugaacu.....................   1      1     LCK-3
..................................................................................................................................................................................................................uuccaAagcuuucuugaacu.....................   3      1     LT-3
..................................................................................................................................................................................................................uuccaAagcuuucuugaacu.....................   7      1     LCK-3
..................................................................................................................................................................................................................uuccacagcuuucuugaacA.....................   2      1     LCK-1
..................................................................................................................................................................................................................uuccacagcuuucuugaaAu.....................   6      1     LCK-3
..................................................................................................................................................................................................................uuccGcagcuuucuugaacu.....................   1      1     LCK-3
..................................................................................................................................................................................................................uuccacagcCuucuugaacu.....................   1      1     JCK-1
..................................................................................................................................................................................................................uuccacaAcuuucuugaacu.....................   1      1     LT-1
..................................................................................................................................................................................................................uuccacagcuuucuugaacG.....................   8      1     LT-2
..................................................................................................................................................................................................................uNccacagcuuucuugaacu.....................   1      1     JT-1
..................................................................................................................................................................................................................uuccacagcuuucuugaaNu.....................   1      1     LT-1
..................................................................................................................................................................................................................uuccacagcuuucuugaacu.....................   3389   0     JCK-2
..................................................................................................................................................................................................................uuUcacagcuuucuugaacu.....................   4      1     JCK-2
..................................................................................................................................................................................................................uuccacagcuuucuugaacA.....................   196    1     LCK-3
..................................................................................................................................................................................................................uuccacagcuuuUuugaacu.....................   1      1     LT-3
..................................................................................................................................................................................................................uuccacagUuuucuugaacu.....................   1      1     LT-1
..................................................................................................................................................................................................................uuccacagcuuucuugaaAu.....................   1      1     JCK-1
..................................................................................................................................................................................................................uuccacagcuuucuugaaAu.....................   1      1     JCK-3
..................................................................................................................................................................................................................uuccCcagcuuucuugaacu.....................   1      1     JCK-2
..................................................................................................................................................................................................................uuccacagcuCucuugaacu.....................   2      1     LT-2
..................................................................................................................................................................................................................uuccacCgcuuucuugaacu.....................   13     1     LT-2
..................................................................................................................................................................................................................uuGcacagcuuucuugaacu.....................   5      1     LT-2
..................................................................................................................................................................................................................uuccacagcuuucuugGacu.....................   8      1     LCK-3
..................................................................................................................................................................................................................uucUacagcuuucuugaacu.....................   3      1     LCK-3
..................................................................................................................................................................................................................uuccacagcuuucuugaacu.....................   2309   0     JCK-3
..................................................................................................................................................................................................................uuccacagcuuucuugaacG.....................   2      1     JCK-2
..................................................................................................................................................................................................................uuccacagcuuGcuugaacu.....................   1      1     JCK-1
..................................................................................................................................................................................................................uuccacCgcuuucuugaacu.....................   3      1     JCK-3
..................................................................................................................................................................................................................uuccacagcuuucuugaacC.....................   9      1     LT-1
..................................................................................................................................................................................................................uCccacagcuuucuugaacu.....................   2      1     JCK-1
..................................................................................................................................................................................................................uuccacagcuuucuugaCcu.....................   25     1     LT-2
..................................................................................................................................................................................................................uuccacagcuuucuugaNcu.....................   1      1     LCK-1
..................................................................................................................................................................................................................uuccacCgcuuucuugaacu.....................   6      1     JCK-2
..................................................................................................................................................................................................................uuccacagcuuucuugaacu.....................   7087   0     LT-3
..................................................................................................................................................................................................................uuccacagcCuucuugaacu.....................   1      1     JT-3
..................................................................................................................................................................................................................uuccacagcuuucuugGacu.....................   1      1     LT-2
..................................................................................................................................................................................................................uuccacagcuuNcuugaacu.....................   3      1     JT-1
..................................................................................................................................................................................................................uNccacagcuuucuugaacu.....................   2      1     LCK-1
..................................................................................................................................................................................................................uuccacagcuuucuugaacC.....................   2      1     LCK-1
..................................................................................................................................................................................................................uuccaAagcuuucuugaacu.....................   1      1     JCK-3
..................................................................................................................................................................................................................uNccacagcuuucuugaacu.....................   1      1     JCK-2
..................................................................................................................................................................................................................uuccacagcuuucuugCacu.....................   3      1     JCK-1
```

Star Mature

uugcuaaaacccacaaauuacuagaaaaaaaaaaaaaguaaagaaaaaaaaaaaaaaaacuuacuacuacuacuaguaguauaucuucuaauuugcuggaaaaggcacaccaaagagaaagaaauuaaaccaaggaagggaggaaggaaggaaggaagugagaucugaugagagaaaaagagaauguuuuugcaaguccuggucaugcuuuuccacagcuuucuugaacuuucuuucuuucuguuuagucu

uuccCcagcuuucuugaacu	3	1	LT-1
uuccacagcuuucuugaacA	57	1	JCK-2
uuccacagcuuuAuugaacu	2	1	JCK-3
uuccUcagcuuucuugaacu	1	1	JCK-2
uuccacagcuuucuugaaUu	52	1	JCK-3
uuccacagcuuucuugaacC	3	1	JT-2
uuccacagcuuuUuugaacu	2	1	LT-1
uuccacagcuuucuugaaAu	2	1	LT-3
uuccacaAcuuucuugaacu	1	1	LCK-2
uuccacagcuuCcuugaacu	1	1	LCK-3
uuccacGgcuuucuugaacu	2	1	JT-3
Cuccacagcuuucuugaacu	2	1	LCK-1
uuccacagcuCucuugaacu	1	1	LCK-3
uuccacagcuuucuugaaUu	18	1	JT-1
uucUacagcuuucuugaacu	1	1	JCK-1
uuccacagcuuucuugaaUu	132	1	LT-1
uuccacagcuuucuugaCcu	3	1	JT-1
uuccacCgcuuucuugaacu	2	1	LCK-1
uuccacagcuuucuugCacu	3	1	JCK-2
uuccacagcuuucuugaacu	2125	0	JCK-1
uuccacagcuuucuugaaUu	72	1	JCK-1
uuccaAagcuuucuugaacu	2	1	JCK-1
uCccacagcuuucuugaacu	1	1	LT-1
uuccaUagcuuucuugaacu	3	1	JCK-2
Cuccacagcuuucuugaacu	1	1	LT-2
uuccacagcuuucuugaacC	3	1	JT-3
uuccCcagcuuucuugaacu	1	1	LCK-1
uuccacaAcuuucuugaacu	1	1	LT-2
uuccacagcuuucuugaCcu	3	1	JCK-1
uuccacagcuuucuuAaacu	3	1	LCK-3
Cuccacagcuuucuugaacu	2	1	LT-1
uuccaAagcuuucuugaacu	3	1	LT-1
uuccacagcuuucuCgaacu	1	1	LT-3
uuccacagcuuucuugCacu	2	1	LCK-2
uuccacagcuuucuugaacG	1	1	JT-1
uuccacagcCuucuugaacu	1	1	LT-3
uCccacagcuuucuugaacu	1	1	LT-3
uuccacCgcuuucuugaacu	8	1	LT-3
uuccacagcuuucuugaGcu	2	1	LCK-3
uuccacagcuuucuugGacu	1	1	LT-3
uuccacagcuuucuugaacC	7	1	JCK-1
uuccacagcuuucuugaacA	137	1	LT-2
uuccacagcuuNcuugaacu	3	1	JCK-1
uuccacagcuuucuugaacu	731	0	JT-3
uuccacagcuuucuugaaAu	6	1	LT-2
uuccacagcuuucCugaacu	1	1	JCK-2
uuccacagcuuucuugaaUu	192	1	LCK-3
uuccacagcCuucuugaacu	7	1	LT-2
uuccacagcuuucCugaacu	2	1	LCK-3
uuccacagcuuucuugaacA	48	1	JCK-1
uuccacagcuuucuugaCcuu	3	1	JCK-1
uuccCcagcuuucuugaacuu	1	1	LCK-3
uuccacagcuuucuugaacNu	2	1	JCK-1
uuccacagcuuucuugaaAuu	1	1	LT-1
uuccacagcuuucuuAaacuu	1	1	JCK-1
uuccacagcuuucuugaaUuu	135	1	LT-3
uuccacagAuuucuugaacuu	1	1	JCK-1
uuccacagcuuucuugaacuu	3888	0	JCK-2
uuccCcagcuuucuugaacuu	5	1	LT-2
uuccacagcuuucuugaaAuu	5	1	LT-2
uuccaUagcuuucuugaacuu	1	1	LT-3
uuccacagcuuucCugaacuu	1	1	JCK-2
uuccacagcuuucuCgaacuu	5	1	LT-2
uucNacagcuuucuugaacuu	1	1	JCK-3
uuccacagcuuucuugaacuC	64	1	JCK-2
uuccacagcuuucuugaaUuu	50	1	JCK-1
uuccacagcuuucuugaacAu	1	1	LT-1
uuccacagcuuucuugaacGu	1	1	JCK-2
uuccacagcuuucuugCacuu	5	1	JCK-2
uuccacagcuuucuugCacuu	2	1	LT-2

```
                                                                                                                                                                                                                  Star                                                                          Mature
uugcuaaaacccacaaauuacuagaaaaaaaaaaaaaguaaagaaaaaaaaaaaaaaaaacuuacuacuacuacuaguaguauaucuucuaauuugcuggaaaaggcacaccaaagagaaagaaauuaaaccaaggaagggaggaaggaaggaaggaagugagaucugaugagagaaaaagagaauguuuuugcaaguccuggucaugcuuuuccacagcuuucuugaacuuucuuucuuucuguuuagucu
..................................................................................................................................................................................................................uuccaAagcuuucuugaacuu....................    1      1    LT-2
..................................................................................................................................................................................................................uuccacagcuuucuugaaUuu....................    88     1    JCK-2
..................................................................................................................................................................................................................uuccacagcuuucuugaacuu....................    1431   0    LT-2
..................................................................................................................................................................................................................uuccacagcuuucuugaacAu....................    5      1    JCK-2
..................................................................................................................................................................................................................uuccacagcuuucuugCacuu....................    1      1    LCK-3
..................................................................................................................................................................................................................uuccacagcuuucuugaaUuu....................    112    1    LCK-3
..................................................................................................................................................................................................................uuUcacagcuuucuugaacuu....................    1      1    JCK-1
..................................................................................................................................................................................................................uuccacagcuuucuugaaUuu....................    3      1    JT-2
..................................................................................................................................................................................................................uuccacCgcuuucuugaacuu....................    3      1    JCK-2
..................................................................................................................................................................................................................uuccacagNuuucuugaacuu....................    1      1    JCK-3
..................................................................................................................................................................................................................uuccacagcuuucuugaacuu....................    2349   0    JCK-1
..................................................................................................................................................................................................................uuccacagcuuucuugaacuA....................    148    1    LT-3
..................................................................................................................................................................................................................uuccaAagcuuucuugaacuu....................    5      1    JCK-2
..................................................................................................................................................................................................................uuccacGgcuuucuugaacuu....................    1      1    JCK-1
..................................................................................................................................................................................................................uuccacaAcuuucuugaacuu....................    1      1    LCK-3
..................................................................................................................................................................................................................uuccacagcuuucuugaacuA....................    2      1    JT-2
..................................................................................................................................................................................................................uCccacagcuuucuugaacuu....................    1      1    LCK-3
..................................................................................................................................................................................................................uuccacagcuuucuugaacuA....................    125    1    JCK-2
..................................................................................................................................................................................................................uuccCcagcuuucuugaacuu....................    2      1    JCK-3
..................................................................................................................................................................................................................uuccacagcuuucuugCacuu....................    2      1    JCK-1
..................................................................................................................................................................................................................uuccaAagcuuucuugaacuu....................    3      1    JCK-3
..................................................................................................................................................................................................................uuccacagcuuucuugaCcuu....................    2      1    JT-1
..................................................................................................................................................................................................................uuccacagcuuucuugaCcuu....................    1      1    LT-3
..................................................................................................................................................................................................................uuccacagcuuucuugaacuN....................    4      1    JCK-1
..................................................................................................................................................................................................................uuccacagcuuucuuAaacuu....................    1      1    LT-1
..................................................................................................................................................................................................................uuccacCgcuuucuugaacuu....................    1      1    LCK-2
..................................................................................................................................................................................................................Cuccacagcuuucuugaacuu....................    1      1    LT-3
..................................................................................................................................................................................................................uuccacagcuCucuugaacuu....................    1      1    JT-2
..................................................................................................................................................................................................................uuccacagcCuucuugaacuu....................    1      1    JCK-3
..................................................................................................................................................................................................................uuccacagcuuucuugaaUuu....................    48     1    LCK-2
..................................................................................................................................................................................................................uuccacagcuuucuugaacAu....................    6      1    LT-3
..................................................................................................................................................................................................................uuccacagcuuucuugaaUuu....................    101    1    LT-2
..................................................................................................................................................................................................................uuccacagcuuuUuugaacuu....................    1      1    JCK-2
..................................................................................................................................................................................................................uuccacagcuuucuugaacuA....................    67     1    JCK-3
..................................................................................................................................................................................................................uuccacagcuuucuugaacAu....................    8      1    LCK-3
..................................................................................................................................................................................................................uuccacagcuuuAuugaacuu....................    1      1    LCK-3
..................................................................................................................................................................................................................uucUacagcuuucuugaacuu....................    1      1    JCK-3
..................................................................................................................................................................................................................uuccacagcuuucuugaacuA....................    97     1    JT-1
..................................................................................................................................................................................................................uuccacagcuuucuugaacuC....................    53     1    LCK-3
..................................................................................................................................................................................................................uuccacagcuuucuugaacuA....................    20     1    LCK-1
..................................................................................................................................................................................................................uuccacGgcuuucuugaacuu....................    7      1    JT-2
..................................................................................................................................................................................................................uuccacagcuuucuugaacuC....................    68     1    JCK-3
..................................................................................................................................................................................................................uucNacagcuuucuugaacuu....................    1      1    JT-1
..................................................................................................................................................................................................................uucNacagcuuucuugaacuu....................    2      1    JCK-1
..................................................................................................................................................................................................................uuccacagcuuucuugaacuu....................    1769   0    LT-3
..................................................................................................................................................................................................................uuccacagcuuucuugaacuC....................    53     1    JCK-1
..................................................................................................................................................................................................................uuccacagcuuucuugaacuC....................    54     1    LT-3
..................................................................................................................................................................................................................uuccaUagcuuucuugaacuu....................    3      1    JCK-3
..................................................................................................................................................................................................................uuccacagcuuuAuugaacuu....................    3      1    JCK-3
..................................................................................................................................................................................................................uuccacagcuuucuugaacAu....................    3      1    JCK-3
..................................................................................................................................................................................................................uuccacagcuuucuugaacuA....................    134    1    LT-2
..................................................................................................................................................................................................................uuccCcagcuuucuugaacuu....................    2      1    JCK-1
..................................................................................................................................................................................................................uNccacagcuuucuugaacuu....................    6      1    JCK-3
..................................................................................................................................................................................................................uuccacagcuuNcuugaacuu....................    3      1    JCK-1
..................................................................................................................................................................................................................uuccacagcuuucuugaacuu....................    954    0    LT-1
..................................................................................................................................................................................................................uuccCcagcuuucuugaacuu....................    6      1    JCK-2
..................................................................................................................................................................................................................uuccacagcuuucuuAaacuu....................    2      1    LCK-3
..................................................................................................................................................................................................................uucGacagcuuucuugaacuu....................    1      1    JCK-1
..................................................................................................................................................................................................................uuccacagcuuucuugaaUuu....................    29     1    LCK-1
..................................................................................................................................................................................................................uuccacagcuuucuugaacGu....................    1      1    LT-1
..................................................................................................................................................................................................................uuccacagcuuucuugaaUuu....................    11     1    JT-3
..................................................................................................................................................................................................................uNccacagcuuucuugaacuu....................    1      1    JCK-1
..................................................................................................................................................................................................................uuUcacagcuuucuugaacuu....................    1      1    JT-3
..................................................................................................................................................................................................................uuccacagcuuucuugaacuA....................    66     1    LT-1
..................................................................................................................................................................................................................uuccacagcuuucuugaacuu....................    157    0    LCK-1
..................................................................................................................................................................................................................uuccacagcuuuUuugaacuu....................    1      1    LT-1
..................................................................................................................................................................................................................Cuccacagcuuucuugaacuu....................    3      1    JCK-3
..................................................................................................................................................................................................................uuccacCgcuuucuugaacuu....................    3      1    LT-3
..................................................................................................................................................................................................................uuccaUagcuuucuugaacuu....................    1      1    JCK-1
..................................................................................................................................................................................................................uCccacagcuuucuugaacuu....................    1      1    LT-2
```

```
Star Mature
uugcuaaaacccacaaauuacuagaaaaaaaaaaaaaguaaagaaaaaaaaaaaaaaaaacuuacuacuacuacuaguaguauaucuucuaauuugcuggaaaaggcacaccaaagagaaagaaauuaaaccaaggaagggaggaaggaaggaaggaagugagaucugaugagagaaaaagagaauguuuuugcaaguccuggucaugcuuuuccacagcuuucuugaacuuucuuucuuucuguuuagucu
...................................................................................................................................................................................................................uuccacCgcuuucuugaacuu.................... 3 1 LCK-3
...................................................................................................................................................................................................................uuUcacagcuuucuugaacuu.................... 2 1 JCK-3
...................................................................................................................................................................................................................uuccacagcuuucuugaacCu.................... 1 1 LT-2
...................................................................................................................................................................................................................uuccacagcuuuAuugaacuu.................... 1 1 LT-3
...................................................................................................................................................................................................................uuccacCgcuuucuugaacuu.................... 5 1 JCK-1
...................................................................................................................................................................................................................uuccacaAcuuucuugaacuu.................... 1 1 LT-2
...................................................................................................................................................................................................................uuccacaAcuuucuugaacuu.................... 1 1 LT-1
...................................................................................................................................................................................................................uuccacagcuuuAuugaacuu.................... 1 1 LT-2
...................................................................................................................................................................................................................uucUacagcuuucuugaacuu.................... 4 1 JCK-2
...................................................................................................................................................................................................................uuccacagcuuCcuugaacuu.................... 1 1 LT-2
...................................................................................................................................................................................................................uuccacagcuuucuugaacuu.................... 191 0 LCK-2
...................................................................................................................................................................................................................uuccacagcuuucuugaacuC.................... 9 1 LCK-2
...................................................................................................................................................................................................................uuccacagcuuucuugaacuA.................... 240 1 LCK-3
...................................................................................................................................................................................................................uuccacagcuuucCugaacuu.................... 1 1 JCK-1
...................................................................................................................................................................................................................uuccacCgcuuucuugaacuu.................... 1 1 JT-1
...................................................................................................................................................................................................................uuccacagcuuucuugaacAu.................... 2 1 JCK-1
...................................................................................................................................................................................................................uuccacagcuuucuugCacuu.................... 1 1 LT-1
...................................................................................................................................................................................................................uuccacagcuuucuugCacuu.................... 2 1 LT-3
...................................................................................................................................................................................................................uuccacagcuuucuugaaUuu.................... 86 1 LT-1
...................................................................................................................................................................................................................uuccaAagcuuucuugaacuu.................... 2 1 JCK-1
...................................................................................................................................................................................................................uCccacagcuuucuugaacuu.................... 1 1 JCK-2
...................................................................................................................................................................................................................uuccacagcuuucuugaacuu.................... 2427 0 LCK-3
...................................................................................................................................................................................................................uuccacagcuuucuugCacuu.................... 1 1 JT-1
...................................................................................................................................................................................................................uuccacGgcuuucuugaacuu.................... 1 1 JCK-2
...................................................................................................................................................................................................................uuUcacagcuuucuugaacuu.................... 6 1 JCK-2
...................................................................................................................................................................................................................uuccacagcuuucuugaacuC.................... 6 1 LCK-1
...................................................................................................................................................................................................................uuccacagcuuucuugaacuA.................... 23 1 LCK-2
...................................................................................................................................................................................................................uuccacagcuuucuugaCcuu.................... 3 1 LCK-3
...................................................................................................................................................................................................................uNccacagcuuucuugaacuu.................... 1 1 LT-2
...................................................................................................................................................................................................................uuUcacagcuuucuugaacuu.................... 2 1 JT-2
...................................................................................................................................................................................................................uuccacGgcuuucuugaacuu.................... 11 1 JT-3
...................................................................................................................................................................................................................uuccacagcuuucuuAaacuu.................... 1 1 LCK-2
...................................................................................................................................................................................................................uuccacagcuuucuugaacGu.................... 1 1 LT-3
...................................................................................................................................................................................................................uuccacCgcuuucuugaacuu.................... 3 1 JCK-3
...................................................................................................................................................................................................................uuccacagcuuucuugaaUuu.................... 61 1 JCK-3
...................................................................................................................................................................................................................uuccacagcuuucuugaacuu.................... 502 0 JT-1
...................................................................................................................................................................................................................uuccacagcuuucuugaacuA.................... 75 1 JCK-1
...................................................................................................................................................................................................................uuccacagcuuucuugaacuu.................... 2955 0 JCK-3
...................................................................................................................................................................................................................uuccacagcuuucCugaacuu.................... 1 1 LT-3
...................................................................................................................................................................................................................uuccacagcuuucuugaCcuu.................... 5 1 JCK-2
...................................................................................................................................................................................................................uuccCcagcuuucuugaacuu.................... 3 1 LT-3
...................................................................................................................................................................................................................uNccacagcuuucuugaacuu.................... 4 1 JCK-2
...................................................................................................................................................................................................................uuccacagcuuucuugCacuu.................... 2 1 JCK-3
...................................................................................................................................................................................................................uuccacagcCuucuugaacuu.................... 2 1 LT-1
...................................................................................................................................................................................................................uuccacaAcuuucuugaacuu.................... 1 1 JCK-3
...................................................................................................................................................................................................................uuccacagcuuucuugaacuC.................... 44 1 LT-2
...................................................................................................................................................................................................................uuccacagcuuucuugaacuu.................... 145 0 JT-3
...................................................................................................................................................................................................................uCccacagcuuucuugaacuu.................... 1 1 LCK-2
...................................................................................................................................................................................................................uuccacagcuuucuugaCcuu.................... 4 1 LT-2
...................................................................................................................................................................................................................uuccacagcuuucuugaCcuu.................... 2 1 JCK-3
...................................................................................................................................................................................................................uuccacagcuuucuugaacAu.................... 6 1 LT-2
...................................................................................................................................................................................................................uuNcacagcuuucuugaacuu.................... 4 1 LT-2
...................................................................................................................................................................................................................uNccacagcuuucuugaacuu.................... 1 1 JT-1
...................................................................................................................................................................................................................uuccacagcuuucuugaacuA.................... 5 1 JT-3
...................................................................................................................................................................................................................uuccacagcuuucuugaGcuu.................... 4 1 JCK-1
...................................................................................................................................................................................................................uuccaNagcuuucuugaacuu.................... 2 1 LCK-1
...................................................................................................................................................................................................................uuccacagcuuucuugaacuu.................... 97 0 JT-2
...................................................................................................................................................................................................................uuccacagcuuucuugaacuC.................... 33 1 LT-1
...................................................................................................................................................................................................................uuccacagcuuucuugaaAuu.................... 3 1 LCK-3
...................................................................................................................................................................................................................uuccacagcuuucuugaacuC.................... 18 1 JT-1
...................................................................................................................................................................................................................uuccacagcuuucuugaCcuuu................... 2 1 LT-3
...................................................................................................................................................................................................................uuccaAagcuuucuugaacuuu................... 1 1 JCK-3
...................................................................................................................................................................................................................uuccacagcuuucuugaCcuuu................... 1 1 LCK-2
...................................................................................................................................................................................................................uuccacagcuuucuugaacuCu................... 3 1 LT-3
...................................................................................................................................................................................................................Cuccacagcuuucuugaacuuu................... 1 1 JCK-1
...................................................................................................................................................................................................................uuccacagcuuucuugCacuuu................... 1 1 LCK-2
...................................................................................................................................................................................................................uuccacagcuuuAuugaacuuu................... 1 1 LT-1
...................................................................................................................................................................................................................uuccacagcuuucuugaacuAu................... 13 1 LT-2
...................................................................................................................................................................................................................uuccacagcuuucuugaacuCu................... 1 1 LT-2
...................................................................................................................................................................................................................uuccacagcuuucuugaacuuu................... 1068 0 LT-1
```

Star Mature

uugcuaaaacccacaaauuacuagaaaaaaaaaaaaaguaaagaaaaaaaaaaaaaaaacuuacuacuacuacuaguaguauaucuucuaauuugcuggaaaaggcacaccaaagagaaagaaauuaaaccaaggaagggaggaaggaaggaaggaagugagaucugaugagagaaaaagagaauguuuuugcaaguccuggucaugcuuuuccacagcuuucuugaacuuucuuucuuucuguuuagucu

uuccacagcuuucuugaacuuu	64	0	JT-3
uuccCcagcuuucuugaacuuu	4	1	LCK-3
uuccacagcuuucuugaaUuuu	39	1	LCK-3
uuccacagcuuucuugaacuuG	2	1	JCK-2
uuccacagcuuucuugaacAuu	29	1	LT-3
uuccaNagcuuucuugaacuuu	1	1	LCK-1
uuccacagcuuuAuugaacuuu	2	1	JCK-1
uuccacagcuCucuugaacuuu	1	1	LT-1
uuccacagcuuucuugaacuuA	49	1	JT-1
uuccacagcuuucuugaacuCu	5	1	LCK-3
uuccacagcuuucuugaacuuu	1108	0	JCK-3
uuccacagcuuucuugCacuuu	2	1	LT-2
uuccacagcuuucuugaaUuuu	27	1	JCK-1
uuccCcagcuuucuugaacuuu	1	1	JCK-3
uuccacagcuuucuugaacuuC	12	1	JT-3
uuccacagcuuucuugaaUuuu	22	1	LCK-1
uuccacagcuuucuugaaAuuu	2	1	LT-3
uuccacCgcuuucuugaacuuu	1	1	LCK-3
uuUcacagcuuucuugaacuuu	1	1	LT-3
uuccacagcuuucuugaacGuu	8	1	JCK-2
uuccacagcCuucuugaacuuu	2	1	JCK-1
uuccacagcuuucuugaaUuuu	63	1	JCK-2
uuccacagcuuucuugaacuuA	50	1	JCK-1
uuccacagcuuucuugaacuCu	5	1	JCK-3
uuccacagcuuucuugaaUuuu	1	1	JT-3
uuccacagcuuucuugaacAuu	4	1	JT-3
uuccacagcuuucuugaacCuu	1	1	LT-3
uuccacaAcuuucuugaacuuu	1	1	JT-3
uuccacagcuuucuugCacuuu	1	1	JCK-3
uuccacagcuuucuugaacuuC	73	1	LT-1
uuccacagcuuucuugaacAuu	16	1	LCK-3
uuccacagcuuuAuugaacuuu	4	1	LT-2
uuccacCgcuuucuugaacuuu	8	1	LT-2
uuccacCgcuuucuugaacuuu	2	1	LT-1
uuccacagcuuucuugaacCuu	3	1	JCK-2
uuccacagcuuucuugaacuuA	157	1	LCK-3
uuccacagcuuucuugaacAuu	6	1	JT-2
uuccacagcuuucuugaaUuuu	32	1	LT-3
uuccacagcuuucuugaacuuA	31	1	LCK-2
uCccacagcuuucuugaacuuu	1	1	LT-1
uuccacagcuGucuugaacuuu	1	1	LT-2
uuccacagcuuucuugaacuuA	10	1	JT-2
uuUcacagcuuucuugaacuuu	7	1	LT-2
uuccacagcuuucuugaacuuC	8	1	JT-2
uuccacagcuuucuugaacuuC	55	1	JCK-3
uuccacagcuuucuugCacuuu	2	1	LT-1
uuccacagcuuucuugaacuuN	1	1	LT-2
uuccCcagcuuucuugaacuuu	2	1	JCK-2
uuccacagcuuucuugaacuuC	89	1	JCK-2
uuccacagcuuucuugaacCuu	1	1	JCK-1
uuccacagcuuucuugaacuuG	7	1	LT-2
uuccacagcuuucuugaCcuuu	3	1	LT-2
uuccCcagcuuucuugaacuuu	3	1	LT-2
uuccacagcuuucuugaacuuA	62	1	LT-1
uNccacagcuuucuugaacuuu	1	1	JCK-3
uuccacagcuuNcuugaacuuu	1	1	JT-1
uuNcacagcuuucuugaacuuu	4	1	LT-2
uuccacagcuuCcuugaacuuu	1	1	LT-2
uuccacaAcuuucuugaacuuu	2	1	LT-2
uNccacagcuuucuugaacuuu	1	1	JCK-1
uuccacagcuuucuugaCcuuu	2	1	JCK-2
uuccacagcuuucuugaacuuu	70	0	JT-2
uuccacagcuuucuugaacuAu	12	1	LT-3
uuccacagcuuucuugaacuCu	1	1	LT-1
Cuccacagcuuucuugaacuuu	1	1	JT-1
uuccacagcuuucuugaacuAu	6	1	JCK-3
uuccacagcuuucuugaacAuu	12	1	LT-1
uuccacCgcuuucuugaacuuu	2	1	LT-3
uuccCcagcuuucuugaacuuu	4	1	LT-3
uucNacagcuuucuugaacuuu	1	1	JCK-1

Star Mature

uugcuaaaacccacaaauuacuagaaaaaaaaaaaaguaaagaaaaaaaaaaaaaaaacuuacuacuacuacuaguaguauaucuucuaauuugcuggaaaaggcacaccaaagagaaagaaauuaaaccaaggaagggaggaaggaaggaaggaagugagaucugaugagagaaaaagagaauguuuuugcaaguccuggucaugcuuuuccacagcuuucuugaacuuucuuucuuucuguuuagucu			
....uuccacagcuuucuugaaUuuu...................	41	1	LT-1
....uCccacagcuuucuugaacuuu...................	1	1	JCK-3
....uuccacagcuuucuugaacuCu...................	2	1	JCK-1
....uuccacagcuuucuugaacAuu...................	12	1	JCK-3
....uuccacagcuuucuugaacuuu...................	1496	0	JCK-2
....uuccacagcuuucuugaaUuuu...................	7	1	JT-1
....uuccaAagcuuucuugaacuuu...................	1	1	JCK-2
....uuccacCgcuuucuugaacuuu...................	2	1	JCK-2
....uuccacagcCuucuugaacuuu...................	2	1	JCK-2
....uuccacCgcuuucuugaacuuu...................	1	1	JT-1
....uuccacagcuuucuugaacuuu...................	377	0	JT-1
....uuccacagcuuucuugaacuAu...................	11	1	LT-1
....uuccacagcuuucuugaCcuuu...................	2	1	LT-1
....uNccacagcuuucuugaacuuu...................	1	1	JCK-2
....uGccacagcuuucuugaacuuu...................	1	1	LCK-3
....uuccacagcuuucuugaacuAu...................	17	1	LCK-3
....uuccacagcuuucuugaacuNu...................	1	1	JT-2
....uuccacagcuuucuugaacGuu...................	3	1	JCK-1
....uuccacagcuGucuugaacuuu...................	2	1	JCK-1
....uuccaUagcuuucuugaacuuu...................	1	1	LT-1
....uuccacagcuuucuugaacCuu...................	1	1	LCK-1
....uuccacCgcuuucuugaacuuu...................	2	1	LCK-2
....uuccacagcuuucCugaacuuu...................	1	1	JCK-2
....uuccacagcuuucuugaacCuu...................	3	1	JCK-3
....Cuccacagcuuucuugaacuuu...................	1	1	LT-3
....uuccacagcuuucuCgaacuuu...................	3	1	LT-3
....Cuccacagcuuucuugaacuuu...................	1	1	JCK-3
....uuccacagcuuucuugaaUuuu...................	37	1	LT-2
....uuccacagcuuucuugaacuCu...................	6	1	JCK-2
....uuccacagcuuucuugaCcuuu...................	1	1	LCK-3
....uuccacagcuuucuugaacuuC...................	41	1	JT-1
....uuccacCgcuuucuugaacuuu...................	3	1	JCK-3
....uuUcacagcuuucuugaacuuu...................	1	1	JCK-2
....uuccacagcCuucuugaacuuu...................	4	1	JCK-3
....uCccacagcuuucuugaacuuu...................	3	1	LT-3
....uuccacagcuuucuugaacGuu...................	1	1	LT-3
....uNccacagcuuucuugaacuuu...................	2	1	LCK-3
....uuccGcagcuuucuugaacuuu...................	1	1	LT-2
....uuccacagcuuucuugaacuuA...................	121	1	LT-3
....uuccacagcuuucuugaacuuC...................	114	1	LT-2
....uuccacagcuuucuugaaUuuu...................	3	1	JT-2
....uuccacagcuuucuugaacuNu...................	1	1	JCK-1
....uuccacagcuuucuugCacuuu...................	1	1	JT-3
....uuccacagcuuucuugaaAuuu...................	2	1	JCK-3
....uuccacagcuuucuugaacuAu...................	16	1	JCK-2
....uuccacagcuuucuugaacuuG...................	2	1	LT-1
....uuUcacagcuuucuugaacuuu...................	1	1	JCK-1
....uuccacagcuuucuugaacuuu...................	250	0	LCK-1
....uuccacagcuuNcuugaacuuu...................	3	1	JCK-1
....uuccacagcuuucuugaacuuu...................	1512	0	LT-3
....uuccacagcuuucuugaacuuu...................	899	0	JCK-1
....uuccacagcuuucuugCacuuu...................	1	1	LCK-3
....uucUacagcuuucuugaacuuu...................	1	1	LCK-3
....uuccacagcuuucuugaaUuuu...................	13	1	LCK-2
....uuccacagcuuucuugaaAuuu...................	3	1	JCK-2
....uuccacagcuuucuugaacAuu...................	26	1	LT-2
....uuccacagcuuucuugaaAuuu...................	2	1	JT-3
....uuccacagcuuucuugaacuuA...................	128	1	JCK-2
....uuccacagcuuucuugaacAuu...................	14	1	JCK-1
....uuccCcagcuuucuugaacuuu...................	1	1	LT-1
....uuccacagcuuucuuUaacuuu...................	1	1	LT-3
....uuccacagcuuucNugaacuuu...................	1	1	LCK-1
....uuccacagcuuucuugaacuuG...................	3	1	LT-3
....uuccacagcuuucuugaacuuC...................	65	1	JCK-1
....uuccacagcuuucuugaacuuu...................	2105	0	LCK-3
....uuccacagcuuGcuugaacuuu...................	2	1	JCK-1
....uuccacagcuuucuugaacuuC...................	253	1	LCK-3
....uuGcacagcuuucuugaacuuu...................	2	1	LT-2
....uuccacagcuuucuugaacuAu...................	3	1	JCK-1
....uuccacCgcuuucuugaacuuu...................	1	1	JCK-1

Star Mature

uugcuaaaacccacaaauuacuagaaaaaaaaaaaaaguaaagaaaaaaaaaaaaaaaacuuacuacuacuacuaguaguauaucuucuaauuugcuggaaaaggcacaccaaagagaaagaaauuaaaccaaggaagggaggaaggaaggaaggaagugagaucugaugagagaaaaagagaauguuuuugcaaguccuggucaugcuuuuccacagcuuucuugaacuuucuuucuuucuguuuagucu

uuccacagcuuucuugaacuuC	118	1	LT-3
uuccacagcuuucuugaacuuN	1	1	JCK-1
uuccacagcuuucuugaacuuu	246	0	LCK-2
uuccacagcCuucuugaacuuu	2	1	LT-2
uuccacagcuuucuugaacuuA	121	1	LT-2
uuccacagcuuucuugaacuuu	1432	0	LT-2
uuccaAagcuuucuugaacuuu	1	1	JCK-1
uuccacagcuuucuugaacAuu	36	1	JCK-2
uuccacagcuuucuugCacuuu	1	1	JCK-1
uuccacagcuuucuugaacuuA	17	1	JT-3
uuccacagcuuucuugaacuuA	21	1	LCK-1
uuccacagcuuucuugaaAuuu	4	1	JCK-1
uuccCcagcuuucuugaacuuu	1	1	JCK-1
uuccacagcuuucuugaacuuA	77	1	JCK-3
uuccacagcuuucuugCacuuu	1	1	JCK-2
uuccacagcuuucuugaaUuuu	36	1	JCK-3
uuccacagcuuucuugaacAuuc	3	1	LT-3
uuccacagcuuucuugaacuuuc	3	0	JT-3
uuccacagcuuucuugaacuuuc	47	0	LT-3
uuccacagcuuucuugaaUuuuc	4	1	LT-1
uuccacagcuuucuugaacuuuU	492	1	JCK-2
uuccacagcuuucuugaacuuuc	16	0	JT-1
uuccacagcuuucuugaacuuuU	376	1	LT-2
uuccacagcuuucuugaacuuuA	16	1	JT-3
uuccacagcuuucuugaacuuuG	9	1	LT-2
uuccacagcuuucuugaacuuCc	1	1	LT-1
uuccacagcuuucuugCacuuuc	1	1	LT-2
uuccacagcuuucuugaacuuuA	40	1	LCK-1
uuccacagcuuucuugaacAuuc	10	1	LCK-3
uuccacCgcuuucuugaacuuuc	1	1	LT-2
uuccacagcuuucuugaacuuuA	11	1	JT-2
uuccacagcuuucuugCacuuuc	1	1	LCK-3
uuccacagcuuucuugaacuuuU	9	1	JT-2
uuccacagcuuucuugaacuuuG	11	1	LT-3
uuccacagcuuucuugaacuuuU	306	1	LT-1
uuccacagcuuucuugaacuuuU	369	1	JCK-3
uuccacagcuuucuugaacuuuU	134	1	LCK-2
uuccacagcuuucuugaacuAuc	4	1	LT-1
uuccacagcuuucuugaacuuuA	230	1	LT-2
uuccacagcuuucuugaacuuuA	47	1	JT-1
uuccacagcuuucuugaacuuAc	5	1	JT-3
uuccacagcuuucuugaacuuuA	144	1	JCK-1
uuccacagcuuucuugaacuuuA	119	1	LT-1
uuccacagcuuucuugaacuuuc	33	0	JCK-1
uuccacagcuuucuugaacuuuG	4	1	JCK-1
uuccacagcuuucuugaacuuuc	40	0	JCK-3
uuccacagcuuucuugaaUuuuc	5	1	JCK-3
uuccacagcuuucuugaacuuuA	48	1	LCK-2
uuccacagcuuucuugaacuuuG	13	1	LT-1
uuccacagcuuucuugaacuuAc	1	1	LT-2
uuccacagcuuucuugaacAuuc	1	1	JCK-1
uuccacagcuuucuugaacuuuc	28	0	LT-1
uuccacagcuuucuugaacuuuc	61	0	LT-2
uuccacagcuuucuugaacuuAc	1	1	JT-2
uuccacagcuuucuugaacAuuc	2	1	LT-2
uuccCcagcuuucuugaacuuuc	1	1	LT-2
uuccacCgcuuucuugaacuuuc	1	1	LCK-3
uuccacagcuuucuugaacuAuc	7	1	LT-2
uuccacagcuuucuugaacAuuc	4	1	LT-1
uuccacagcuuucuugaacuuuG	9	1	LCK-3
uuccacagcuuucuugaacuuuA	166	1	LT-3
uuccacagcuuucuugaaUuuuc	6	1	LCK-3
uuccacagcuuucuugaacuuAc	1	1	JCK-3
uuccacagcuuucuugaacuuuU	101	1	JT-1
uuccacagcuuucuugaacuuuU	18	1	JT-3
uuccacagcuuucuugaacuuuU	324	1	LT-3
uuccacagcuuucuugaaUuuuc	15	1	LCK-1
uuccacagcuuucuugaaUuuuc	13	1	LCK-2
uuccacagcuuucuugaacuuuc	50	0	JCK-2
uuccacagcuuucuugaacAuuc	1	1	JCK-3

```
                                                                                                                                                                                                                  Star                                             Mature
uugcuaaaacccacaaauuacuagaaaaaaaaaaaaaguaaagaaaaaaaaaaaaaaaacuuacuacuacuacuaguaguauaucuucuaauuugcuggaaaaggcacaccaaagagaaagaaauuaaaccaaggaagggaggaaggaaggaaggaagugagaucugaugagagaaaaagagaauguuuuugcaaguccuggucaugcuuuuccacagcuuucuugaacuuucuuucuuucuguuuagucu
..................................................................................................................................................................................................................uuccacagcuuucuugaacuuuU..................  490  1  LCK-3
..................................................................................................................................................................................................................uuccacagcuuucuugaacuAuc..................  1    1  LT-3
..................................................................................................................................................................................................................uuccacagcuuucuugaacuuuA..................  177  1  JCK-2
..................................................................................................................................................................................................................uuccacagcuuucuugaacuuuU..................  294  1  JCK-1
..................................................................................................................................................................................................................uuccacagcuuucuugaacuuuc..................  3    0  JT-2
..................................................................................................................................................................................................................uuccacagcuuucuugaacuuAc..................  1    1  LT-3
..................................................................................................................................................................................................................uuccacagcuuucuugaacuuuG..................  8    1  JCK-2
..................................................................................................................................................................................................................uuccacGgcuuucuugaacuuuc..................  1    1  LT-2
..................................................................................................................................................................................................................uuccacagcuuucuugaacuuuc..................  97   0  LCK-3
..................................................................................................................................................................................................................uuccacagcuuucuugaaUuuuc..................  3    1  JCK-2
..................................................................................................................................................................................................................uuccacagcuuucuugaacuuuA..................  155  1  JCK-3
..................................................................................................................................................................................................................uuNcacagcuuucuugaacuuuc..................  1    1  LT-2
..................................................................................................................................................................................................................uuccacagcuuucuugaacuuuA..................  301  1  LCK-3
..................................................................................................................................................................................................................uuccacagcuuucuugaacuuuG..................  4    1  JCK-3
..................................................................................................................................................................................................................uuccacagcuuucuugaaAuuuc..................  1    1  LT-3
..................................................................................................................................................................................................................uuccacagcuuucuugaacuuuU..................  140  1  LCK-1
..................................................................................................................................................................................................................uuccacagcuuucuugaacuuuUu.................  15   1  LT-2
..................................................................................................................................................................................................................uuccacagcuuucuugaacuuucA.................  4    1  JCK-3
..................................................................................................................................................................................................................uuccacagcuuucuugaacuuuAu.................  2    1  JCK-1
..................................................................................................................................................................................................................uuccacagcuuucuugaacuAucu.................  1    1  JCK-1
..................................................................................................................................................................................................................uuccacagcuuucuugaacuuuUu.................  89   1  JCK-2
..................................................................................................................................................................................................................uuccacagcuuucuugaacuAucu.................  4    1  JCK-2
..................................................................................................................................................................................................................uuccacagcuuucuugaacuuucA.................  1    1  LT-1
..................................................................................................................................................................................................................uuccacagcuuucuugaacuuuAu.................  4    1  JT-1
..................................................................................................................................................................................................................uuccacagcuuucuugaacuAucu.................  1    1  JCK-3
..................................................................................................................................................................................................................uuccacagcuuucuugaacuuucu.................  3    0  LT-1
..................................................................................................................................................................................................................uuccacagcuuucuugaacuuucA.................  6    1  JT-1
..................................................................................................................................................................................................................uuccacagcuuucuugaacuuAcu.................  1    1  LT-1
..................................................................................................................................................................................................................uuccacagcuuucuugaacuuuAu.................  5    1  LCK-3
..................................................................................................................................................................................................................uuccacagcuuucuugaacuuuAu.................  6    1  LT-3
..................................................................................................................................................................................................................uuccacagcuuucuugaacuuucA.................  1    1  LT-3
..................................................................................................................................................................................................................uuccacagcuuucuugaacuAucu.................  5    1  LCK-3
..................................................................................................................................................................................................................uuccacagcuuucuugaacuuucA.................  2    1  JCK-2
..................................................................................................................................................................................................................uuccacagcuuucuugaacuuuUu.................  49   1  JCK-3
..................................................................................................................................................................................................................uuccacagcuuucuugaacuuuUu.................  19   1  LT-1
..................................................................................................................................................................................................................uuccacagcuuucuugaacuuucA.................  6    1  LT-2
..................................................................................................................................................................................................................uuccacagcuuucuugaacuuuAu.................  2    1  LT-2
..................................................................................................................................................................................................................uuccacagcuuucuugaacuuAcu.................  2    1  JCK-2
..................................................................................................................................................................................................................uuccacagcuuucuugaacuuuUu.................  13   1  LCK-3
..................................................................................................................................................................................................................uuccacagcuuucuugaacuuuAu.................  6    1  JCK-2
..................................................................................................................................................................................................................uuccacagcuuucuugaacuuCcu.................  1    1  LCK-3
..................................................................................................................................................................................................................uuccacagcuuucuugaacuuuAu.................  2    1  LT-1
..................................................................................................................................................................................................................uuccacagcuuucuugaacuuuUu.................  47   1  JCK-1
..................................................................................................................................................................................................................uuccacagcuuucuugaacuuAcu.................  2    1  LT-2
..................................................................................................................................................................................................................uuccacagcuuucuugaacuuucu.................  2    0  LCK-3
..................................................................................................................................................................................................................uuccacagcuuucuugaacuuuUu.................  13   1  LT-3
..................................................................................................................................................................................................................uuccacagcuuucuugaacuuuAu.................  7    1  JCK-3
..................................................................................................................................................................................................................uuccacagcuuucuugaacuuucA.................  1    1  JCK-1
..................................................................................................................................................................................................................uuccacagcuuucuugaacuuAcu.................  1    1  LT-3
..................................................................................................................................................................................................................uuccacagcuuucuugaacuuuUuu................  9    1  JCK-1
..................................................................................................................................................................................................................uuccacagcuuucuugaacuuucuA................  1    1  LT-2
..................................................................................................................................................................................................................uuccacagcuuucuugaacuuuAuu................  12   1  JCK-3
..................................................................................................................................................................................................................uuccacagcuuucuugaacuuuAuu................  7    1  JCK-1
..................................................................................................................................................................................................................uuccacagcuuucuugaacuuuUuu................  2    1  LCK-3
..................................................................................................................................................................................................................uuccacagcuuucuugaacuuucuC................  1    1  LT-3
..................................................................................................................................................................................................................uuccacagcuuucuugaacuuuUuu................  6    1  JCK-3
..................................................................................................................................................................................................................uuccacagcuuucuugaacuuuAuu................  7    1  LCK-3
..................................................................................................................................................................................................................uuccacagcuuucuugaacuuuUuu................  2    1  LT-3
..................................................................................................................................................................................................................uuccacagcuuucuugaacuuuUuu................  5    1  LT-2
..................................................................................................................................................................................................................uuccacagcuuucuugaacuuuAuu................  3    1  LT-3
..................................................................................................................................................................................................................uuccacagcuuucuugaacuuAcuu................  3    1  JCK-2
..................................................................................................................................................................................................................uuccacagcuuucuugaacuuuGuu................  1    1  LT-3
..................................................................................................................................................................................................................uuccacagcuuucuugaacuuuAuu................  4    1  LT-2
..................................................................................................................................................................................................................uuccacagcuuucuugaacuuuAuu................  8    1  JCK-2
..................................................................................................................................................................................................................uuccacagcuuucuugaacuuuUuu................  1    1  LT-1
..................................................................................................................................................................................................................uuccacagcuuucuugaacuuuAuu................  5    1  LT-1
..................................................................................................................................................................................................................uuccacagcuuucuugaacuuuUuu................  12   1  JCK-2
..................................................................................................................................................................................................................uuccacagcuuucuugaacuuuAuuu...............  3    1  LCK-3
..................................................................................................................................................................................................................uuccacagcuuucuugaacuuuAuuu...............  3    1  JCK-2
..................................................................................................................................................................................................................uuccacagcuuucuugaacuuuAuuu...............  3    1  JCK-1
```

```
                                                                                                                                                 Star                                                          Mature
uugcuaaaacccacaaauuacuagaaaaaaaaaaaaaguaaagaaaaaaaaaaaaaaaacuuacuacuacuacuaguaguauaucuucuaauuugcuggaaaaggcacaccaaagagaaagaaauuaaaccaaggaagggaggaaggaaggaaggaagugagaucugaugagagaaaaagagaaauguuuuugcaagucuggucaugcuuuuccacagcuuucuugaacuuucuuucuuucuguuuagucu
..................................................................................................................................................................................................................uuccacagcuuucuugaacuuuAuuu...............    5    1    JCK-3
..................................................................................................................................................................................................................uuccacagcuuucuugaacuuuUuuu...............    2    1    LCK-3
..................................................................................................................................................................................................................uuccacagcuuucuugaacuuucuuu...............    1    0    JCK-1
...................................................................................................................................................................................................................uccacagcuuucuugaac......................    4    0    LT-1
...................................................................................................................................................................................................................uccacagcuuucuugaac......................    2    0    JCK-2
...................................................................................................................................................................................................................uccacagcuuucuugaac......................    2    0    JCK-3
...................................................................................................................................................................................................................Cccacagcuuucuugaac......................    1    1    LT-1
...................................................................................................................................................................................................................uccacagcuuucuugaac......................    2    0    JT-2
...................................................................................................................................................................................................................uccacagcuuucuugaac......................    4    0    JT-3
...................................................................................................................................................................................................................uccacagcuCucuugaac......................    1    1    LT-3
...................................................................................................................................................................................................................uccacagcuuucuugaac......................    10   0    LT-2
...................................................................................................................................................................................................................uccacaUcuuucuugaac......................    1    1    LT-3
...................................................................................................................................................................................................................uccacagcuuucuugaac......................    6    0    JT-1
...................................................................................................................................................................................................................uccacagcuuucuugaac......................    13   0    LCK-3
...................................................................................................................................................................................................................ucNacagcuuucuugaac......................    1    1    LT-2
...................................................................................................................................................................................................................uccacagcuuucuugaac......................    11   0    LT-3
...................................................................................................................................................................................................................uccacagcuuucuugaacu.....................    10   0    JCK-2
...................................................................................................................................................................................................................uccacagcuuucuugaacu.....................    29   0    LCK-2
...................................................................................................................................................................................................................uccacagcuuucuugaaUu.....................    1    1    LT-1
...................................................................................................................................................................................................................uccacagcuuucuugaacu.....................    7    0    LT-1
...................................................................................................................................................................................................................uccacagcuuucuugaaAu.....................    3    1    LT-2
...................................................................................................................................................................................................................uccacagcuuucuugaacA.....................    1    1    LT-3
...................................................................................................................................................................................................................uccacagcuuucuugaacu.....................    53   0    LCK-1
...................................................................................................................................................................................................................uccacagcuuucuugaacu.....................    12   0    LCK-3
...................................................................................................................................................................................................................uccacagcuuucuugaacA.....................    1    1    LT-1
...................................................................................................................................................................................................................uccacagcuuucuugaacu.....................    18   0    LT-3
...................................................................................................................................................................................................................uccacagcuuucuugaacu.....................    2    0    JCK-1
...................................................................................................................................................................................................................uccacagcuuucuugaacu.....................    2    0    LT-2
...................................................................................................................................................................................................................uccacagcuuucuugaacu.....................    7    0    JCK-3
...................................................................................................................................................................................................................uccacagcuuucuugaacuu....................    2    0    LT-1
...................................................................................................................................................................................................................uccacagcuuucuugaacuu....................    1    0    JCK-1
...................................................................................................................................................................................................................uccacagcuuucuugaacuu....................    7    0    LT-2
...................................................................................................................................................................................................................uccacagcuuucuugaacuu....................    2    0    LT-3
...................................................................................................................................................................................................................uccacagcuuucuugaacuu....................    6    0    JCK-2
...................................................................................................................................................................................................................uccacagcuuucuugaacuu....................    5    0    JCK-3
...................................................................................................................................................................................................................uccacagcuuucuugaacuu....................    9    0    LCK-3
...................................................................................................................................................................................................................uccacagcuuucuugaaUuu....................    1    1    LT-3
...................................................................................................................................................................................................................Cccacagcuuucuugaacuuu...................    1    1    JCK-1
...................................................................................................................................................................................................................uccacagcuuucuugaacuuu...................    2    0    JCK-1
...................................................................................................................................................................................................................uccacagcuuucuugaacuuu...................    5    0    LT-2
...................................................................................................................................................................................................................uccacagcuuucuugaacuuu...................    4    0    LT-3
...................................................................................................................................................................................................................uccacagcuuucuugaacuuu...................    1    0    JCK-2
...................................................................................................................................................................................................................uccacagcuuucuugaacuuu...................    4    0    LCK-3
...................................................................................................................................................................................................................uccacagcuuucuugaacuuu...................    2    0    LT-1
...................................................................................................................................................................................................................uccacagcuuucuugaacuuC...................    1    1    LT-2
...................................................................................................................................................................................................................uccacagcuuucuugaacAuu...................    4    1    LT-2
...................................................................................................................................................................................................................uccacagcuuucuugaacuuuU..................    6    1    LCK-3
...................................................................................................................................................................................................................uccacagcuuucuugaacuuuA..................    1    1    LT-2
...................................................................................................................................................................................................................uccacagcuuucuugaacuuuc..................    2    0    LT-1
...................................................................................................................................................................................................................uccacagcuuucuugaacuuuc..................    1    0    JCK-2
...................................................................................................................................................................................................................uccacagcuuucuugaacuuuU..................    1    1    JCK-1
...................................................................................................................................................................................................................uccacagcuuucuugaacuuuc..................    1    0    JCK-1
...................................................................................................................................................................................................................uccacagcuuucuugaacuuuA..................    10   1    LCK-3
...................................................................................................................................................................................................................uccacagcuuucuugaacuuuU..................    2    1    LT-3
...................................................................................................................................................................................................................uccacagcuuucuugaacuuuU..................    3    1    LT-1
...................................................................................................................................................................................................................uccacagcuuucuugaacuuuU..................    3    1    JCK-2
...................................................................................................................................................................................................................uccacagcuuucuugaacuuuU..................    2    1    JCK-3
...................................................................................................................................................................................................................uccacagcuuucuugaacuuuU..................    1    1    LT-2
...................................................................................................................................................................................................................uccacagcuuucuugaacuuuUu.................    2    1    LCK-3
....................................................................................................................................................................................................................ccacagcuuucuugaacu.....................    36   0    LCK-2
....................................................................................................................................................................................................................ccacagcuuucuugaacu.....................    318  0    LCK-3
....................................................................................................................................................................................................................ccacagcuuucuugaacu.....................    14   0    JT-2
....................................................................................................................................................................................................................ccacagcuuucuugaacu.....................    51   0    JCK-1
....................................................................................................................................................................................................................ccacagcuuucuugaaUu.....................    5    1    LT-3
....................................................................................................................................................................................................................ccacagcuuuAuugaacu.....................    1    1    LT-3
....................................................................................................................................................................................................................ccacagcuuucuugaaUu.....................    2    1    LCK-3
....................................................................................................................................................................................................................ccacagcuuucuugCacu.....................    2    1    LT-3
....................................................................................................................................................................................................................ccacagcuuucuugaaUu.....................    2    1    LT-1
....................................................................................................................................................................................................................ccCcagcuuucuugaacu.....................    1    1    LT-2
....................................................................................................................................................................................................................ccacagcCuucuugaacu.....................    1    1    LT-3
```

Star Mature

```
uugcuaaaacccacaaauuacuagaaaaaaaaaaaaguaaagaaaaaaaaaaaaaaaacuuacuacuacuacuaguaguauaucuucuaauuugcuggaaaaggcacaccaaagagaaagaaauuaaaccaaggaagggaggaaggaaggaaggaagugagaucugaugagagaaaaagagaauguuuuugcaaguccuggucaugcuuuuccacagcuuucuugaacuuucuuucuuucuguuuagucu
...................................................................................................................................................................................................................ccacagcuuucuugaacu.....................	301	0	LT-2
...................................................................................................................................................................................................................ccacagcuuucuugaacA.....................	1	1	JCK-1
...................................................................................................................................................................................................................ccacagcuuucuugaCcu.....................	1	1	LT-3
...................................................................................................................................................................................................................ccacagcuuucuugaCcu.....................	1	1	JT-1
...................................................................................................................................................................................................................ccacagcuuucuugaacu.....................	24	0	LCK-1
...................................................................................................................................................................................................................ccacagcuuucuugaacC.....................	1	1	LT-3
...................................................................................................................................................................................................................ccacagcuuucuugaacu.....................	89	0	JCK-2
...................................................................................................................................................................................................................Acacagcuuucuugaacu.....................	1	1	LT-2
...................................................................................................................................................................................................................ccacagcuuucuugaCcu.....................	2	1	LCK-3
...................................................................................................................................................................................................................ccacagcuuucuugaacG.....................	1	1	LT-3
...................................................................................................................................................................................................................ccacCgcuuucuugaacu.....................	1	1	LT-3
...................................................................................................................................................................................................................ccacagcuuucuugaacu.....................	51	0	JT-1
...................................................................................................................................................................................................................ccacagcuuucuugaacu.....................	69	0	JCK-3
...................................................................................................................................................................................................................ccacagcuuucuugaacA.....................	2	1	LT-2
...................................................................................................................................................................................................................ccacagcuuucuugaacA.....................	1	1	LT-3
...................................................................................................................................................................................................................ccacagcuuucuugaacu.....................	20	0	JT-3
...................................................................................................................................................................................................................ccacagcuuuUuugaacu.....................	4	1	LT-3
...................................................................................................................................................................................................................ccacagcuuucuugaacC.....................	2	1	JCK-1
...................................................................................................................................................................................................................ccacagcuuucuugaacu.....................	342	0	LT-3
...................................................................................................................................................................................................................ccacagcuuucuugaacu.....................	104	0	LT-1
...................................................................................................................................................................................................................ccacagcuuucuugaacA.....................	1	1	JCK-2
...................................................................................................................................................................................................................ccacagcuuucuugaacuA....................	41	1	LCK-3
...................................................................................................................................................................................................................ccacagcuuucuugaacuC....................	5	1	LT-1
...................................................................................................................................................................................................................ccacagcuuucuugaacuu....................	251	0	LT-3
...................................................................................................................................................................................................................ccacagcuuucuugaacuu....................	42	0	JT-3
...................................................................................................................................................................................................................ccacagcuuucuugaacuA....................	11	1	JCK-1
...................................................................................................................................................................................................................ccacagcuuucuugaacuu....................	68	0	JT-1
...................................................................................................................................................................................................................ccacagcuuucuugCacuu....................	1	1	JCK-2
...................................................................................................................................................................................................................ccacCgcuuucuugaacuu....................	1	1	JCK-2
...................................................................................................................................................................................................................ccacagcuuucuugaacuA....................	6	1	LT-1
...................................................................................................................................................................................................................ccacagcuuucuugaacuA....................	19	1	LT-2
...................................................................................................................................................................................................................ccacagcuuucuugaacuA....................	3	1	JT-2
...................................................................................................................................................................................................................ccacagcuuucuugaacuu....................	252	0	JCK-2
...................................................................................................................................................................................................................cNacagcuuucuugaacuu....................	1	1	JCK-2
...................................................................................................................................................................................................................ccacagcuuucuugaacuC....................	2	1	JCK-1
...................................................................................................................................................................................................................ccacagcuuucuugaacAu....................	1	1	JCK-1
...................................................................................................................................................................................................................ccacagcuuucuugaacuA....................	9	1	LT-3
...................................................................................................................................................................................................................ccacagcuuucuugCacuu....................	2	1	JCK-3
...................................................................................................................................................................................................................ccacagcuuucuugaacuC....................	10	1	JT-1
...................................................................................................................................................................................................................ccacagcuuucuugaaUuu....................	3	1	JCK-1
...................................................................................................................................................................................................................ccacagcuuucuugaacuu....................	34	0	JT-2
...................................................................................................................................................................................................................ccacagcuuucuugaacuA....................	17	1	JCK-2
...................................................................................................................................................................................................................ccacagcuuucuugaCcuu....................	1	1	LCK-3
...................................................................................................................................................................................................................ccacagcuuucuugaacuu....................	66	0	LT-1
...................................................................................................................................................................................................................ccacagcuuucuugaCcuu....................	1	1	LT-2
...................................................................................................................................................................................................................ccacagcuuucuugaacuu....................	180	0	JCK-3
...................................................................................................................................................................................................................ccacagcuuucuugaaUuu....................	6	1	LCK-3
...................................................................................................................................................................................................................ccacCgcuuucuugaacuu....................	2	1	LCK-3
...................................................................................................................................................................................................................ccacagcuuucuugaacuu....................	246	0	LT-2
...................................................................................................................................................................................................................ccacagcuuucuugaaAuu....................	1	1	JCK-2
...................................................................................................................................................................................................................ccacagcuuucuugaaUuu....................	5	1	JCK-2
...................................................................................................................................................................................................................ccacagcuuucuugaacuA....................	10	1	JT-1
...................................................................................................................................................................................................................cNacagcuuucuugaacuu....................	1	1	JCK-3
...................................................................................................................................................................................................................ccacCgcuuucuugaacuu....................	1	1	JT-1
...................................................................................................................................................................................................................ccacagcuuucuugaacuC....................	12	1	JCK-2
...................................................................................................................................................................................................................ccacagcuuucuugCacuu....................	2	1	LT-2
...................................................................................................................................................................................................................ccacagcuuucuugaacuu....................	168	0	JCK-1
...................................................................................................................................................................................................................ccacagcuuucuugaaUuu....................	9	1	LT-2
...................................................................................................................................................................................................................ccacagcuuucuugaacCu....................	1	1	LCK-3
...................................................................................................................................................................................................................ccacagcuuucNugaacuu....................	1	1	JCK-1
...................................................................................................................................................................................................................ccCcagcuuucuugaacuu....................	1	1	LT-3
...................................................................................................................................................................................................................ccacagcuuucuugaacuC....................	3	1	LT-2
...................................................................................................................................................................................................................ccacagcuuucuugaaAuu....................	3	1	LT-3
...................................................................................................................................................................................................................ccacagcuuucuugaacuu....................	349	0	LCK-3
...................................................................................................................................................................................................................ccacagcuuucuugaaUuu....................	7	1	LT-3
...................................................................................................................................................................................................................ccaAagcuuucuugaacuu....................	1	1	JCK-1
...................................................................................................................................................................................................................ccacagcuuucuugaaUuu....................	3	1	LT-1
...................................................................................................................................................................................................................cNacagcuuucuugaacuu....................	1	1	JT-1
...................................................................................................................................................................................................................ccacagcuuucuugaacuC....................	4	1	JCK-3
...................................................................................................................................................................................................................ccacagcuuucuugaacuA....................	8	1	JCK-3
```

```
                                                                                                                                                                       Star                                                                        Mature
uugcuaaaacccacaaauuacuagaaaaaaaaaaaaaguaaagaaaaaaaaaaaaaaaacuuacuacuacuacuaguaguauaucuucuaauuugcuggaaaaggcacaccaaagagaaagaaauuaaaccaaggaagggaggaaggaaggaaggaagugagaucugaugagagaaaaagagaauguuuuugcaaguccuggucaugcuuuuccacagcuuucuugaacuuucuuucuuucuguuuagucu
....................................................................................................................................................................................................................ccacagcuuucuugaCcuu....................	1	1	JT-2
....................................................................................................................................................................................................................ccacagcuuucuugaaUuu....................	4	1	JCK-3
....................................................................................................................................................................................................................ccacagcuuucuugaacuA....................	5	1	JT-3
....................................................................................................................................................................................................................ccacagcuuucuugaacuC....................	4	1	LT-3
....................................................................................................................................................................................................................ccaNagcuuucuugaacuu....................	1	1	JCK-1
....................................................................................................................................................................................................................ccacagcCuucuugaacuu....................	3	1	LCK-3
....................................................................................................................................................................................................................ccacagcuuucuugaaUuuu...................	27	1	LCK-1
....................................................................................................................................................................................................................ccacagcuuucuugaacAuu...................	1	1	JCK-2
....................................................................................................................................................................................................................ccacagcuuucuugaacuuA...................	41	1	JCK-1
....................................................................................................................................................................................................................ccacCgcuuucuugaacuuu...................	1	1	JT-1
....................................................................................................................................................................................................................ccacagcuuucuugaacuuu...................	495	0	JCK-2
....................................................................................................................................................................................................................ccacagcuuucuugaaUuuu...................	4	1	JCK-1
....................................................................................................................................................................................................................ccacagcuuucuugaacuuu...................	485	0	LT-2
....................................................................................................................................................................................................................ccacagcuuucuugaacAuu...................	5	1	LCK-3
....................................................................................................................................................................................................................ccacagcuuucuugaacuuu...................	77	0	LCK-1
....................................................................................................................................................................................................................ccacagcuuucuugaacuCu...................	4	1	LT-1
....................................................................................................................................................................................................................ccNcagcuuucuugaacuuu...................	1	1	LCK-1
....................................................................................................................................................................................................................ccacagcuuucuugaacuuA...................	51	1	LT-2
....................................................................................................................................................................................................................ccacagcCuucuugaacuuu...................	1	1	JCK-3
....................................................................................................................................................................................................................ccacagcuuucuugaacAuu...................	2	1	JCK-1
....................................................................................................................................................................................................................ccacagcuuucuugaacuuG...................	1	1	LT-3
....................................................................................................................................................................................................................ccacagcuuucuugaacuuG...................	2	1	JCK-2
....................................................................................................................................................................................................................ccacagcuuucuugaacuuu...................	31	0	JT-3
....................................................................................................................................................................................................................ccacagcuuucuugaacuuG...................	1	1	JCK-3
....................................................................................................................................................................................................................ccacagcuuucuugaacuuA...................	10	1	LT-1
....................................................................................................................................................................................................................ccacagcuuucuugaacuuC...................	4	1	LT-1
....................................................................................................................................................................................................................ccacagcuuucuugaaUuuu...................	7	1	LT-3
....................................................................................................................................................................................................................ccacagcuuucuugaacuAu...................	5	1	JCK-3
....................................................................................................................................................................................................................ccacCgcuuucuugaacuuu...................	3	1	JCK-3
....................................................................................................................................................................................................................ccacagcuuucuugaacuuu...................	506	0	LCK-3
....................................................................................................................................................................................................................ccacagcuuucuugaacAuu...................	2	1	LT-1
....................................................................................................................................................................................................................ccacagGuuucuugaacuuu...................	1	1	JT-3
....................................................................................................................................................................................................................ccacagcuuucuugaacGuu...................	1	1	JCK-1
....................................................................................................................................................................................................................ccacagcuuucuugaaUuuu...................	13	1	LCK-3
....................................................................................................................................................................................................................ccacagcuuucuugaacuAu...................	16	1	LT-2
....................................................................................................................................................................................................................ccacagcuuucuugaacuuu...................	169	0	LT-1
....................................................................................................................................................................................................................ccacagcuuucuugaacuuu...................	334	0	JCK-1
....................................................................................................................................................................................................................ccacagcuuucuugaaUuuu...................	18	1	LCK-2
....................................................................................................................................................................................................................ccacagcuuucuugNacuuu...................	1	1	LCK-1
....................................................................................................................................................................................................................ccacagcuuucuugaCcuuu...................	1	1	JCK-3
....................................................................................................................................................................................................................ccacagcuuucuugaacuuA...................	53	1	JCK-3
....................................................................................................................................................................................................................Ucacagcuuucuugaacuuu...................	1	1	JCK-1
....................................................................................................................................................................................................................ccacagcuuucuugaacuuA...................	40	1	LCK-3
....................................................................................................................................................................................................................ccacagcuuucuuAaacuuu...................	1	1	LT-2
....................................................................................................................................................................................................................ccacagcuuucuugaacuuu...................	71	0	LCK-2
....................................................................................................................................................................................................................ccacagcuuucuugaacuCu...................	7	1	JCK-3
....................................................................................................................................................................................................................ccacagcuuCcuugaacuuu...................	1	1	LT-3
....................................................................................................................................................................................................................ccacagcuuucuugaacuCu...................	4	1	JCK-1
....................................................................................................................................................................................................................ccacagcuuucuugaacuuC...................	15	1	JCK-1
....................................................................................................................................................................................................................ccacagcuuucuugaacAuu...................	3	1	LT-2
....................................................................................................................................................................................................................ccacagcuuucuugaacuCu...................	4	1	LT-2
....................................................................................................................................................................................................................ccacagcuuucuugaacuCu...................	17	1	JCK-2
....................................................................................................................................................................................................................ccacagcuuucuugaacuuC...................	14	1	LT-2
....................................................................................................................................................................................................................ccacagcuuucuugaacuuC...................	5	1	LCK-3
....................................................................................................................................................................................................................ccacagcuuucuugaGcuuu...................	1	1	LCK-2
....................................................................................................................................................................................................................ccacagcuuucuugCacuuu...................	1	1	JCK-2
....................................................................................................................................................................................................................ccacCgcuuucuugaacuuu...................	1	1	JCK-1
....................................................................................................................................................................................................................Ucacagcuuucuugaacuuu...................	4	1	JCK-2
....................................................................................................................................................................................................................ccacagcuuucuugaacuuA...................	69	1	JCK-2
....................................................................................................................................................................................................................ccacagcuuucuugCacuuu...................	1	1	LT-2
....................................................................................................................................................................................................................ccacagcuuucuugaacuuC...................	24	1	JCK-2
....................................................................................................................................................................................................................ccacaNcuuucuugaacuuu...................	1	1	LCK-1
....................................................................................................................................................................................................................ccacagcuuucuugaacuuC...................	20	1	LT-3
....................................................................................................................................................................................................................ccacagcuuucuugaacuuu...................	349	0	JCK-3
....................................................................................................................................................................................................................ccacagcuuucuugaacuAu...................	6	1	JCK-1
....................................................................................................................................................................................................................ccaAagcuuucuugaacuuu...................	1	1	LCK-1
....................................................................................................................................................................................................................ccacagcuuucuugaacuAu...................	9	1	LT-3
....................................................................................................................................................................................................................ccacaAcuuucuugaacuuu...................	1	1	JCK-3
....................................................................................................................................................................................................................cNacagcuuucuugaacuuu...................	1	1	JCK-1
....................................................................................................................................................................................................................ccacagcuuucuugaacuuG...................	1	1	LT-1
```

```
                                                                                                                                                 Star                                                          Mature
uugcuaaaacccacaaauuacuagaaaaaaaaaaaaaguaaagaaaaaaaaaaaaaaaacuuacuacuacuacuaguaguauaucuucuaauuugcuggaaaaggcacaccaaagagaaagaaauuaaaccaaggaagggaggaaggaaggaaggaagugagaucugaugagagaaaaagagaauguuuuugcaaguccuggucaugcuuuuccacagcuuucuugaacuuucuuucuuucuguuuagucu
....................................................................................................................................................................................................................ccacagcuuucuugaacuuG...................   7     1    JCK-1
....................................................................................................................................................................................................................ccacagcuuucuugaacAuu...................   2     1    LT-3
....................................................................................................................................................................................................................ccacagcuuucuugaacuAu...................   9     1    LCK-3
....................................................................................................................................................................................................................ccacagcuuucuugaacuuu...................   43    0    JT-1
....................................................................................................................................................................................................................ccacagcuuucuugaacuuC...................   18    1    JCK-3
....................................................................................................................................................................................................................ccacagcuuucuugaacuuu...................   439   0    LT-3
....................................................................................................................................................................................................................ccacagcuuucuugaacuuA...................   48    1    LT-3
....................................................................................................................................................................................................................ccacagcuuucuugCacuuu...................   1     1    LCK-3
....................................................................................................................................................................................................................ccacagcuuucuugaacuCu...................   8     1    LT-3
....................................................................................................................................................................................................................ccacCgcuuucuugaacuuu...................   2     1    LT-2
....................................................................................................................................................................................................................ccacagcuuucuugaacuuA...................   11    1    JT-2
....................................................................................................................................................................................................................ccacagcuuucuugaacuAu...................   9     1    JCK-2
....................................................................................................................................................................................................................ccacagcuuucuugaaUuuu...................   3     1    JCK-3
....................................................................................................................................................................................................................ccacagcuuucuugaacuuA...................   8     1    JT-3
....................................................................................................................................................................................................................ccacagcuuucuugaacuuu...................   21    0    JT-2
....................................................................................................................................................................................................................ccacagcuuucuugaaAuuu...................   5     1    LCK-3
....................................................................................................................................................................................................................ccacagcuuucuugaacuuAc..................   1     1    LT-2
....................................................................................................................................................................................................................ccacagcuuucuugaacuuuc..................   29    0    LCK-3
....................................................................................................................................................................................................................ccacagcuuucuugaacuuuU..................   2     1    JT-2
....................................................................................................................................................................................................................ccacagcuuucuugaacuuuU..................   64    1    LCK-2
....................................................................................................................................................................................................................ccacagcuuucuugaacuuCc..................   1     1    LT-3
....................................................................................................................................................................................................................ccacagcuuucuugaacuuuU..................   153   1    JCK-3
....................................................................................................................................................................................................................ccacagcuuucuugaacuuuG..................   17    1    LCK-3
....................................................................................................................................................................................................................ccacagcuuucuugaacuuuU..................   87    1    LT-1
....................................................................................................................................................................................................................ccacagcuuucuugaCcuuuc..................   1     1    LT-1
....................................................................................................................................................................................................................ccacagcuuucuugaacuuuG..................   6     1    LT-3
....................................................................................................................................................................................................................ccacagcuuucuugaacuuuU..................   223   1    LT-3
....................................................................................................................................................................................................................ccacagcuuucuugaacuuCc..................   1     1    LCK-3
....................................................................................................................................................................................................................ccacagcuuucuugaacuuCc..................   1     1    JCK-1
....................................................................................................................................................................................................................ccacagcuuucuugaacuuuG..................   14    1    LT-2
....................................................................................................................................................................................................................ccacagcuuucuugaacuuuA..................   178   1    JCK-2
....................................................................................................................................................................................................................ccacagcuuucuugaacuuuU..................   349   1    LCK-3
....................................................................................................................................................................................................................ccacagcuuucuugaacuuuc..................   27    0    LCK-1
....................................................................................................................................................................................................................ccacagcuuucuugaacuuuU..................   52    1    LCK-1
....................................................................................................................................................................................................................ccacagcuuucuugaacuuuc..................   8     0    JCK-2
....................................................................................................................................................................................................................ccacagcuuucuugaacuuuA..................   109   1    LCK-3
....................................................................................................................................................................................................................ccacagcuuucuugaacuuuc..................   3     0    LT-1
....................................................................................................................................................................................................................ccacagcuuucuugaacuuuG..................   6     1    LT-1
....................................................................................................................................................................................................................ccacagcuuucuugaacuuuG..................   10    1    JCK-3
....................................................................................................................................................................................................................ccacagcuuucuugaacuuuU..................   184   1    JCK-2
....................................................................................................................................................................................................................ccacagcuuucuugaacuuuc..................   30    0    LCK-2
....................................................................................................................................................................................................................ccacagcuuucuugaacuuAc..................   1     1    JCK-2
....................................................................................................................................................................................................................ccacagcuuucuugaacuuuG..................   8     1    JCK-2
....................................................................................................................................................................................................................ccacagcuuucuugaacuuuA..................   90    1    JCK-3
....................................................................................................................................................................................................................ccacagcuuucuugaacuuuA..................   146   1    LT-2
....................................................................................................................................................................................................................ccacagcuuucuugaacuuAc..................   5     1    LCK-3
....................................................................................................................................................................................................................ccacagcuuucuugaacuuuA..................   150   1    JCK-1
....................................................................................................................................................................................................................ccacagcuuucuugaacuuuc..................   9     0    JCK-3
....................................................................................................................................................................................................................ccacagcuuucuugaacuuuA..................   33    1    LT-1
....................................................................................................................................................................................................................ccacagcuuucuugaacuuuA..................   7     1    JT-1
....................................................................................................................................................................................................................ccacagcuuucuugaaUuuuc..................   1     1    LT-2
....................................................................................................................................................................................................................ccacagcuuucuugaacuuuc..................   8     0    LT-3
....................................................................................................................................................................................................................ccacagcuuucuugaacuAuc..................   7     1    LCK-3
....................................................................................................................................................................................................................ccacagcuuucuugaacuAuc..................   1     1    LT-3
....................................................................................................................................................................................................................ccacagcuuucuugaacuuAc..................   3     1    LT-3
....................................................................................................................................................................................................................ccacagcuuucuugaacuuuU..................   13    1    JT-3
....................................................................................................................................................................................................................ccacagcuuucuugaacuuuc..................   4     0    JCK-1
....................................................................................................................................................................................................................ccacagcuuucuugaacuuuG..................   9     1    JCK-1
....................................................................................................................................................................................................................ccacagcuuucuugaacuuuU..................   246   1    LT-2
....................................................................................................................................................................................................................ccacagcuuucuugaacuuuU..................   138   1    JCK-1
....................................................................................................................................................................................................................ccacagcuuucuugaacuuuc..................   16    0    LT-2
....................................................................................................................................................................................................................ccacagcuuucuugaacuuuU..................   42    1    JT-1
....................................................................................................................................................................................................................ccacagcuuucuugaacuuuA..................   82    1    LT-3
....................................................................................................................................................................................................................ccacagcuuucuugaacuuAcu.................   3     1    LT-2
....................................................................................................................................................................................................................ccacagcuuucuugaacuuuUu.................   4     1    JT-3
....................................................................................................................................................................................................................ccacagcuuucuugaacuuucG.................   1     1    JCK-3
....................................................................................................................................................................................................................ccacagcuuucuugaacuuAcu.................   23    1    LCK-3
....................................................................................................................................................................................................................ccacagcuuucuugaacuuucu.................   3     0    LT-3
....................................................................................................................................................................................................................ccacagcuuucuugaacuuucu.................   4     0    JCK-2
....................................................................................................................................................................................................................ccacagcuuucuugaacuuucu.................   4     0    JCK-3
```

```
                                                                                                                                                 Star                                              Mature
uugcuaaaacccacaaauuacuagaaaaaaaaaaaaaguaaagaaaaaaaaaaaaaaaacuuacuacuacuacuaguaguauaucuucuaauuugcuggaaaaggcacaccaaagagaaagaaauuaaaccaaggaagggaggaaggaaggaaggaagugagaucugaugagagaaaaagagaauguuuuugcaaguccuggucaugcuuuuccacagcuuucuugaacuuucuuucuuucuguuuagucu
....................................................................................................................................................................................................................ccacagcuuucuugaacuuuGu.................    5    1    LCK-3
....................................................................................................................................................................................................................ccacagcuuucuugaacuuuAu.................    16   1    LT-2
....................................................................................................................................................................................................................ccacagcuuucuugaacuuucA.................    3    1    LT-3
....................................................................................................................................................................................................................ccacagcuuucuugaacuuuUu.................    38   1    JCK-3
....................................................................................................................................................................................................................ccacagcuuucuugaacuuAcu.................    4    1    JCK-3
....................................................................................................................................................................................................................ccacagcuuucuugaacuuucC.................    2    1    LT-3
....................................................................................................................................................................................................................ccacagcuuucuugaacuuuUu.................    29   1    JCK-1
....................................................................................................................................................................................................................ccacagcuuucuugaacuuAcu.................    4    1    JCK-2
....................................................................................................................................................................................................................ccacagcuuucuugaacuuuUu.................    7    1    LT-1
....................................................................................................................................................................................................................ccacagcuuucuugaacuuuUu.................    22   1    LCK-3
....................................................................................................................................................................................................................ccacagcuuucuugaacuuucu.................    1    0    LT-1
....................................................................................................................................................................................................................ccacagcuuucuugaacuuuAu.................    13   1    LT-3
....................................................................................................................................................................................................................ccacagcuuucuugaacuuuAu.................    23   1    LCK-3
....................................................................................................................................................................................................................ccacagcuuucuugaacuuuAu.................    19   1    JCK-1
....................................................................................................................................................................................................................ccacagcuuucuugaacuAucu.................    2    1    LCK-3
....................................................................................................................................................................................................................ccacagcuuucuugaacuuuAu.................    4    1    LT-1
....................................................................................................................................................................................................................ccacagcuuucuugaacuuuAu.................    1    1    JT-3
....................................................................................................................................................................................................................ccacagcuuucuugaacuuuUu.................    39   1    LT-2
....................................................................................................................................................................................................................ccacagcuuucuugaacuuucu.................    1    0    JCK-1
....................................................................................................................................................................................................................ccacagcuuucuugaacuuAcu.................    1    1    LT-1
....................................................................................................................................................................................................................ccacagcuuucuugaacuuAcu.................    1    1    JCK-1
....................................................................................................................................................................................................................ccacagcuuucuugaacuuucA.................    5    1    JCK-2
....................................................................................................................................................................................................................ccacagcuuucuugaacuuuUu.................    54   1    JCK-2
....................................................................................................................................................................................................................ccacagcuuucuugaacuuuGu.................    3    1    LT-1
....................................................................................................................................................................................................................ccacagcuuucuugaacuuuUu.................    25   1    LT-3
....................................................................................................................................................................................................................ccacagcuuucuugaacuuAcu.................    2    1    LT-3
....................................................................................................................................................................................................................ccacagcuuucuugaacuuuAu.................    16   1    JCK-3
....................................................................................................................................................................................................................ccacagcuuucuugaacuuucA.................    3    1    JCK-3
....................................................................................................................................................................................................................ccacagcuuucuugaacuuuUu.................    2    1    JT-2
....................................................................................................................................................................................................................ccacagcuuucuugaacuuucA.................    1    1    JCK-1
....................................................................................................................................................................................................................ccacagcuuucuugaacuuuAu.................    23   1    JCK-2
....................................................................................................................................................................................................................ccacagcuuucuugaacuuucG.................    5    1    JCK-2
....................................................................................................................................................................................................................ccacagcuuucuugaacuuucA.................    4    1    LT-2
....................................................................................................................................................................................................................ccacagcuuucuugaacuuuAuu................    13   1    JCK-3
....................................................................................................................................................................................................................ccacagcuuucuugaacuuuGuu................    1    1    JCK-2
....................................................................................................................................................................................................................ccacagcuuucuugaacuuuUuu................    5    1    LT-1
....................................................................................................................................................................................................................ccacagcuuucuugaacuuuAuu................    25   1    LCK-3
....................................................................................................................................................................................................................ccacagcuuucuugaacuuucCu................    1    1    JCK-2
....................................................................................................................................................................................................................ccacagcuuucuugaacuuuUuu................    5    1    LT-2
....................................................................................................................................................................................................................ccacagcuuucuugaacuuuAuu................    13   1    LT-2
....................................................................................................................................................................................................................ccacagcuuucuugaacuuuUuu................    11   1    JCK-2
....................................................................................................................................................................................................................ccacagcuuucuugaacuuucAu................    1    1    JCK-2
....................................................................................................................................................................................................................ccacagcuuucuugaacuuucAu................    1    1    JCK-3
....................................................................................................................................................................................................................ccacagcuuucuugaacuuucuA................    2    1    LCK-3
....................................................................................................................................................................................................................ccacagcuuucuugaacuuucuu................    1    0    JCK-3
....................................................................................................................................................................................................................ccacagcuuucuugaacuuuUuu................    4    1    LT-3
....................................................................................................................................................................................................................ccacagcuuucuugaacuuuUuu................    9    1    JCK-3
....................................................................................................................................................................................................................ccacagcuuucuugaacuuuAuu................    3    1    LT-1
....................................................................................................................................................................................................................ccacagcuuucuugaacuuuAuu................    1    1    JT-2
....................................................................................................................................................................................................................ccacagcuuucuugaacuuuAuu................    21   1    JCK-2
....................................................................................................................................................................................................................ccacagcuuucuugaacuuuAuu................    4    1    LT-3
....................................................................................................................................................................................................................ccacagcuuucuugaacuuucuA................    1    1    LT-2
....................................................................................................................................................................................................................ccacagcuuucuugaacuuuAuu................    14   1    JCK-1
....................................................................................................................................................................................................................ccacagcuuucuugaacuuuUuu................    4    1    JCK-1
....................................................................................................................................................................................................................ccacagcuuucuugaacuuuUuu................    14   1    LCK-3
....................................................................................................................................................................................................................ccacagcuuucuugaacuuuAuu................    4    1    JT-3
....................................................................................................................................................................................................................ccacagcuuucuugaacuuuAuuu...............    2    1    JCK-2
....................................................................................................................................................................................................................ccacagcuuucuugaacuuuUuuu...............    3    1    JCK-3
....................................................................................................................................................................................................................ccacagcuuucuugaacuuucAuu...............    1    1    JCK-2
....................................................................................................................................................................................................................ccacagcuuucuugaacuuuAuuu...............    1    1    LT-1
....................................................................................................................................................................................................................ccacagcuuucuugaacuuuUuuu...............    3    1    LT-3
....................................................................................................................................................................................................................ccacagcuuucuugaacuuuAuuu...............    1    1    LT-2
....................................................................................................................................................................................................................ccacagcuuucuugaacuuuAuuu...............    1    1    LT-3
....................................................................................................................................................................................................................ccacagcuuucuugaacuuuAuuu...............    3    1    JCK-1
....................................................................................................................................................................................................................ccacagcuuucuugaacuuucuCu...............    1    1    JCK-2
....................................................................................................................................................................................................................ccacagcuuucuugaacuuuUuuu...............    2    1    JCK-1
....................................................................................................................................................................................................................ccacagcuuucuugaacuuuAuuu...............    1    1    JCK-3
....................................................................................................................................................................................................................ccacagcuuucuugaacuuuUuuu...............    2    1    JCK-2
....................................................................................................................................................................................................................ccacagcuuucuugaacuuuUuuu...............    1    1    LT-2
.....................................................................................................................................................................................................................cacagcuuucuugaacuu....................    3    0    LT-2
```

```
                                                                                                                                                    Star                                                                   Mature
uugcuaaaacccacaaauuacuagaaaaaaaaaaaaaguaaagaaaaaaaaaaaaaaaacuuacuacuacuacuaguaguauaucuucuaauuugcuggaaaaggcacaccaaagagaaagaaauuaaaccaaggaagggaggaaggaaggaaggaagugagaucugaugagagaaaaagagaauguuuuugcaaguccuggucaugcuuuuccacagcuuucuugaacuuucuuucuuucuguuuagucu
.....................................................................................................................................................................................................................cacagcuuucuugaacuu....................    5    0    LCK-3
.....................................................................................................................................................................................................................cacagcuuucuugaacuu....................    1    0    LT-3
.....................................................................................................................................................................................................................cacagcuuucuugaacuu....................    3    0    JCK-3
.....................................................................................................................................................................................................................cCcagcuuucuugaacuu....................    1    1    LCK-3
.....................................................................................................................................................................................................................cacagcuuucuugaacuuu...................    2    0    JCK-3
.....................................................................................................................................................................................................................cacagcuuucuugaacuuu...................    10   0    JT-3
.....................................................................................................................................................................................................................cacagcuuucuugaacuuu...................    4    0    LT-2
.....................................................................................................................................................................................................................cacagcuuucuugaacuuu...................    1    0    JCK-1
.....................................................................................................................................................................................................................cacagcuuucuugaacuuA...................    1    1    JCK-1
.....................................................................................................................................................................................................................cacagcuuucuugaacuuu...................    1    0    JT-2
.....................................................................................................................................................................................................................cacagcuuucuugaacuuA...................    2    1    JCK-3
.....................................................................................................................................................................................................................cacagcuuucuugaacuuA...................    1    1    LT-3
.....................................................................................................................................................................................................................cacagcuuucuugaacuuu...................    6    0    LT-3
.....................................................................................................................................................................................................................cacagcuuucuugaacuuu...................    8    0    JCK-2
.....................................................................................................................................................................................................................cacagcuuucuugaacuuu...................    2    0    LT-1
.....................................................................................................................................................................................................................cacagcuuucuugaacuAu...................    2    1    LT-2
.....................................................................................................................................................................................................................cacagcuuucuugaacuuuA..................    1    1    JCK-3
.....................................................................................................................................................................................................................cacagcuuucuugaacuuuU..................    3    1    JCK-1
.....................................................................................................................................................................................................................cacagcuuucuugaacuuuU..................    5    1    JCK-3
.....................................................................................................................................................................................................................cacagcuuucuugaacuuuU..................    5    1    JCK-2
.....................................................................................................................................................................................................................cacagcuuucuugaacuuuA..................    1    1    LT-3
.....................................................................................................................................................................................................................cacagcuuucuugaacuuuU..................    2    1    LT-2
.....................................................................................................................................................................................................................cacagcuuucuugaacuuuU..................    1    1    LT-1
.....................................................................................................................................................................................................................cacagcuuucuugaacuuuA..................    4    1    LCK-3
.....................................................................................................................................................................................................................cacagcuuucuugaacuuuU..................    2    1    LT-3
.....................................................................................................................................................................................................................cacagcuuucuugaacuuuU..................    5    1    LCK-3
.....................................................................................................................................................................................................................cacagcuuucuugaacuuucu.................    2    0    JCK-2
.....................................................................................................................................................................................................................cacagcuuucuugaacuuucu.................    9    0    JCK-3
.....................................................................................................................................................................................................................cacagcuuucuugaacuuucu.................    3    0    LT-1
.....................................................................................................................................................................................................................cacagcuuucuugaacuuucu.................    2    0    JCK-1
.....................................................................................................................................................................................................................cacagcuuucuugaacuuuUuu................    1    1    LT-1
.....................................................................................................................................................................................................................cacagcuuucuugaacuuuAuu................    2    1    JCK-3
.....................................................................................................................................................................................................................cacagcuuucuugaacuuuUuu................    2    1    LT-3
.....................................................................................................................................................................................................................cacagcuuucuugaacuuuAuu................    3    1    JCK-1
.....................................................................................................................................................................................................................cacagcuuucuugaacuuuAuu................    8    1    JCK-2
.....................................................................................................................................................................................................................cacagcuuucuugaacuuAcuuu...............    1    1    LT-1
......................................................................................................................................................................................................................acagcuuucuugaacuuu...................    1    0    LT-2
......................................................................................................................................................................................................................acagcuuucuugaaUuuu...................    6    1    LCK-3
......................................................................................................................................................................................................................acagcuuucuugaacuuA...................    2    1    LT-2
......................................................................................................................................................................................................................acagcuuucuugaacuuuU..................    1    1    LT-3
......................................................................................................................................................................................................................acagcuuucuugaacuuuU..................    4    1    LCK-3
......................................................................................................................................................................................................................acagcuuucuugaacuuuA..................    1    1    LT-1
......................................................................................................................................................................................................................acagcuuucuugaacuuuU..................    3    1    LT-2
......................................................................................................................................................................................................................acagcuuucuugaacuuuUu.................    3    1    LT-3
......................................................................................................................................................................................................................................uucuGucuuucuguuua....    1    1    LT-2
```
